# Comparative FISH analysis of *Senna tora* tandem repeats revealed insights into the chromosome dynamics in *Senna*

**DOI:** 10.1007/s13258-021-01051-w

**Published:** 2021-03-03

**Authors:** Thanh Dat Ta, Nomar Espinosa Waminal, Thi Hong Nguyen, Remnyl Joyce Pellerin, Hyun Hee Kim

**Affiliations:** Department of Chemistry and Life Science, Bioscience Institute, Sahmyook University, Seoul, 01795 Republic of Korea

**Keywords:** *Senna*, Dysploidy, Repetitive elements, Tandem repeats, FISH, Chromosome rearrangements

## Abstract

**Background:**

DNA tandem repeats (TRs) are often abundant and occupy discrete regions in eukaryotic genomes. These TRs often cause or generate chromosomal rearrangements, which, in turn, drive chromosome evolution and speciation. Tracing the chromosomal distribution of TRs could therefore provide insights into the chromosome dynamics and speciation among closely related taxa. The basic chromosome number in the genus *Senna* is 2*n* = 28, but dysploid species like *Senna tora* have also been observed.

**Objective:**

To understand the dynamics of these TRs and their impact on *S. tora* dysploidization.

**Methods:**

We performed a comparative fluorescence *in situ* hybridization (FISH) analysis among nine closely related *Senna* species and compared the chromosomal distribution of these repeats from a cytotaxonomic perspective by using the ITS1-5.8S-ITS2 sequence to infer phylogenetic relationships.

**Results:**

Of the nine *S. tora* TRs, two did not show any FISH signal whereas seven TRs showed similar and contrasting patterns to other *Senna* species. StoTR01_86, which was localized in the pericentromeric regions in all *S. tora*, but not at the nucleolar organizer region (NOR) site, was colocalized at the NOR site in all species except in *S. siamea*. StoTR02_7_tel was mostly localized at chromosome termini, but some species had an interstitial telomeric repeat in a few chromosomes. StoTR05_180 was distributed in the subtelomeric region in most species and was highly amplified in the pericentromeric region in some species. StoTR06_159 was either absent or colocalized in the NOR site in some species, and StoIGS_463, which was localized at the NOR site in *S. tora*, was either absent or localized at the subtelomeric or pericentromeric regions in other species.

**Conclusions:**

These data suggest that TRs play important roles in *S. tora* dysploidy and suggest the involvement of 45S rDNA intergenic spacers in “carrying” repeats during genome reshuffling.

**Supplementary Information:**

The online version contains supplementary material available at 10.1007/s13258-021-01051-w.

## Introduction

Repetitive elements (REs) comprise a considerable portion of plant genomes, even comprising > 85 % in some plant genomes (Schnable et al. 2009). Although considered ‘junk’ in the past, REs are now known as important players in genome function and structure, and species evolution (Fedoroff 2012; Wicker et al. 2007). REs are generated or produced by chromosomal rearrangements, which drive chromosome structure variations between closely related lineages (Murat et al. 2017; Schubert and Lysak 2011). Tracking the dynamics of various repeat families could therefore provide insights into the genome history of closely related taxa (Long et al. 2013; Waminal et al. 2018b).

REs can be categorized into two classes: tandem repeats (TRs) and dispersed repeats (Kubis et al. 1998). TRs follow a head-to-tail organization, whereas direct repeats, such as transposable elements. TRs can be further classified into three groups based on repeat unit length: microsatellites (2–5 bp repeats), minisatellites (6–100 bp repeats) and satellite DNA (satDNAs) (150–400 bp monomer length) (Mehrotra and Goyal 2014).

TRs are often distributed in distinct chromosomal regions, usually at pericentromeric and subtelomeric sites (Sharma et al. 2013), often in low copies forming a “library” of repeats (Fedoroff and Bennetzen 2013; Ruiz-Ruano et al. 2016). Due to sequence homologies in these chromosomal regions, they have been considered as hotspots for chromosomal rearrangements during genomic perturbations such as those resulting from a genome merger (Hartley and O’Neill 2019; Rosato et al. 2018; Schubert and Lysak 2011).

Often, these chromosomal rearrangements could amplify a single or few TR families, and when reproductive barriers are formed, new species may develop with an altered chromosomal number, organization, or TR abundance (Mandáková and Lysak 2018; Murat et al. 2017). Several basic chromosome numbers are present in a genus, which was described as dysploid (Winterfeld et al. 2020). Ascending dysploids, or species with more chromosomes, are formed when chromosomes are fragmented and when fragments maintain or develop centromeres. In contrast, descending dysploids are formed when chromosomes fuse (Winterfeld et al. 2020). As the differential abundance and distribution of TRs among species in a taxonomic group can vary (Perumal et al. 2017), TRs are used as cytotaxonomic markers to study phylogenetic relationships (Guerra 2008, 2012).

Fluorescence *in situ* hybridization (FISH) of TRs can provide essential information for understanding genome structure, chromosome evolution and phylogenetic relationships among related taxa (Iovene et al. 2008; Matsuda and Chapman 1995). This information complements the inference of phylogenetic relationships based on DNA markers such as the internal transcribed spacers (ITS) of 45S rDNA (Kim et al. 2015), which are used extensively because of their abundance of phylogenetically informative sites and easy amplification in the plant species (Farah et al. 2018). In addition, the intergenic spacers (IGS) of the 45S rDNA coding genes are known to “carry” different TRs during species evolution (Falquet et al. 1997; Almeida et al. 2012). Comparing the distribution of duplicated sequences in the *S. tora* IGS could provide important data for studying *Senna* evolution.

The genus *Senna*, formerly *Cassia* (family Fabaceae, subfamily Caesalpinioideae) comprises approximately 350 species of herbs, shrubs, and trees (Tucker 1996; Monkheang et al. 2011). *Senna* has varied economic and medicinal applications, and has been used not only as a natural pesticide but also for treating skin diseases, gastrointestinal disorders, and inflammation (Ongchai et al. 2019; Singh et al. 2013). A desire to further exploit these health benefits has prompted the genome sequencing of *Senna tora*, as a widespread and representative species, to better understand *Senna* biology and evolution.

The predominant chromosome number of the genus *Senna* is 2*n* = 28 (Rice et al. 2015), but species with descending dysploid karyotypes of 2*n* = 22–26 have also been identified, such as *S. tora* with 2*n* = 26 (Cordeiro and Felix 2018; Pellerin et al. 2019). To understand the dysploidization in *S. tora*, we examined its genome TR composition in our previous work (Waminal et al. 2021). We identified eight *S. tora* TRs, many of which showed unusual chromosomal distributions. For example, StoTR02_7_tel, which is the *Arabidopsis*-type telomeric repeat, showed highly amplified loci in the pericentromeric regions in all *S. tora* chromosomes in addition to signals at chromosome termini. Besides, StoTR05_180, which was observed in the subtelomeric region of all *S. occidentalis* chromosomes was absent in these regions but highly amplified in the pericentromeric regions in *S. tora* chromosomes.

The classification of *Senna* is not yet fully understood, and no comparative cytogenetics have been performed to examine chromosome evolution in the genus. Here, to understand the dynamics of *S. tora* TRs and their role in *S. tora* dysploidy, we performed comparative FISH among nine *Senna* species. We analyzed the chromosomal distribution patterns of these TRs in a cyto-phylogenetic context based on the ITS1-5.8S-ITS2 sequences.

## Materials and methods

### Plant material

Root tips were collected from germinated seeds of nine *Senna* species, which were provided by the National Plant Germplasm System (USDA, USA), Department of Herbal Crop Research (NIHHS, RDA, Korea), and Rare Palm Seeds (Germany). Roots were treated with 2 mM 8-hydroxyquinoline for 5 h at 18 °C to arrest cells at metaphase, and then fixed in Carnoy’s solution and stored in 70 % ethanol until used for chromosome preparation.

### Chromosome preparation

Chromosome spreads were prepared according to Waminal et al. (2012) and Eliazar et al. (2019). Briefly, the meristematic tips were immersed in an enzyme solution consisting of 1 % pectolyase Y-23 (Duchefa Biochemie, Netherlands) and 2 % cellulase R-10 (Duchefa Biochemie, Netherlands) for 60–90 min at 37 °C. Next, chilled Carnoy’s solution was added, and after centrifugation, the precipitate was pipetted and dropped onto pre-warmed glass slides in a humid chamber at 70 °C and then air-dried.

### Probes preparation and fluorescence in situ hybridization (FISH)

*S. tora* TRs were identified using low-coverage sequences and short-read clustering with TAREAN (Novák et al. 2017) in our previous study (Waminal et al. 2018a, 2021). All pre-labeled oligonucleotide probes (PLOPs) used in this study are listed in Table 1.

**Table 1:** List of pre-labeled oligonucleotide probes used in this study

Name	Oligo name	PLOP sequences (5′–3′)	Length (bp)	Modification	References
StoTR01_86	StoTR01_86_OP1	TTAATCAGTTTTCGCCGATGAGTGTTTCG	29	5′-FAM	Waminal et al. (2021)
	StoTR01_86_OP2	CATCAGTTTTCGCCAATGAGTGTTTCG	27		
StoTR02_7_tel	Tel_UniOP_*Arabidopsis*	TTTAGGGTTTAGGGTTTAGGGTTTAGGGT	29	ATTO425	Waminal et al. (2018a)
StoTR03_178	StoTR03_178_OP1	CCGGAATATGTTAAGACATGATCCACGCT	29	5′-Cy5	Waminal et al. (2021)
	StoTR03_178_OP2	ATCTCAGAAACCTTCACGAATTACGAGGC	29		
	StoTR03_178_OP3	CCGGAGTGGTTTTGATGCTCCAATTGGA	28		
StoTR04_55	StoTR04_55_OP	GCGAAAACTGATTAAAAAAAGAAAAATGAATATCAAG	37	5′-AMCA	Waminal et al. (2021)
StoTR05_180	StoTR05_180_OP1	GATTTAATGCTCGAATGGGGCTCGTGATC	29	5′-Texas Red	Waminal et al. (2021)
	StoTR05_180_OP2	GTTGTTGCACAAGTGAGTCAAACCGATC	28		
	StoTR05_180_OP3	TGTTTAGACATGACTTGACACACCTTCCA	29		
	StoTR05_180_OP4	TGAGTTCTTTTGAGATTCAATCGCGATTT	29		
StoTR06_159	StoTR06_159_OP1	TGCATATGCTGGGTCAAAATGAAGCCTAT	29	5′-Cy3	Waminal et al. (2021)
	StoTR06_159_OP2	AGGCTTCCTTGTGTCATAGGCTTCATTTT	29		
StoIGS_463	StoIGS_463_PLOP1	AAACCAATATATATTCTATTTTTCGTGATT	30	5′-FAM	Waminal et al. (2021)
	StoIGS_463_PLOP2	CAAATGATTGATAAGCCTTTAATTTTATTA	30		
	StoIGS_463_PLOP3	GAAATTTTGGGGTTAAGCTTATATATTTTT	30		
Sto_45S_CDS	18SrDNA_UniOP_1	CCGGAGAGGGAGCCTGAGAAACGGCTAC	28	5′-Cy3	Waminal et al. (2018a)
	18SrDNA_UniOP_2	ATCCAAGGAAGGCAGCAGGCGCGCAA	26		
	18SrDNA_UniOP_3	GGGCAAGTCTGGTGCCAGCAGCCGCGGT	28		
	18SrDNA_UniOP_4	TCGAAGACGATYAGATACCGTCSTAGT	27		
	18SrDNA_UniOP_5	CTGAAACTTAAAGGAATTGACGGAAGG	27		
	18SrDNA_UniOP_6	GGAGCCTGCGGCTTAATTTGACTCAAC	27		
	18SrDNA_UniOP_7	GGTGGTGCATGGCCGTTCTTAGTTGGTGG	29		
	18SrDNA_UniOP_8	ACGTCCCTGCCCTTTGTACACACCGCCCGTC	31		
	5.8SrDNA_UniOP_1	AAYGACTCTCGGCAACGGATATCTMG	26		
	5.8SrDNA_UniOP_2	CWYGCATCGATGAAGAACGTAGCRA	25		
	5.8SrDNA_UniOP_3	GCGATACTTGGTGTGAATTGCAGAATC	27		
	5.8SrDNA_UniOP_4	GTGAACCATCGAGTYTTTGAACGCAAGT	28		
Sto_5S	5SrDNA_ang_1	GGATGCGATCATACCAGCACTAAAGCACCG	30	5′-Alexa Fluor 488	Waminal et al. (2018a)
	5SrDNA_gym_1	GRGTGCGATMATACCASCGYTWRYGYA	27		
	5SrDNA_cranial_1	GYYTAYRGCCAYACCACCCTGRRHRCG	27		
	5SrDNA_ang_2	CCCATCAGAACTCCGAAGTTAAGCGTGCT	29		
	5SrDNA_gym_2	ATCCSATCAGAACTCCGYARTTAAGCR	27		
	5SrDNA_cranial_2	GATCTCGTCYGATCTCGGAAGCTAAGC	27		
	5SrDNA_ang_3	GCGAGAGTAGTACTAGGATGGGTG	24		
	5SrDNA_gym_3	TTGGGYYRGAGTAGTACTRGGATGGGT	27		
	5SrDNA_cranial_3	GTCGGGCCYGGTYAGTACTTGGATGGG	27		
	5SrDNA_ang_4	CCTGGGAAGTMCTCGTGTTGCAYYCC	26		
	5SrDNA_gym_4	CTCYYGGGAAGTCCYRRTRTYGCACCC	27		
	5SrDNA_cranial_4	CYGCCTGGGAATACCRGGTGYYGTARG	27		

For the FISH procedure, a total of 40 µL hybridization mixture containing 100 % formamide, 50 % dextran sulfate, 20× SSC, 50 % dextran sulfate, 20× SSC, 50 ng/µL of each probe, and Sigma water were added to each slide and then denatured at 80 °C and kept in a humid chamber at 37 °C overnight. After hybridization, the slides were washed with 2× SSC at room temperature (RT) and then dehydrated through ethanol 70 %, 90 %, and 100 % for 3 min each. Finally, the slides were counterstained with a DAPI-Vectashield solution and captured under Cytovision ver 7.2 software with an Olympus BX53 fluorescence microscope system, equipped with a Leica DFC365 FS CCD camera. The images were enhanced using Adobe Photoshop CS6. Chromosomes were measured using the IdeoKar 1.2 software (Mirzaghaderi and Marzangi 2015), and chromosome typing was performed according to the method of Levan et al. (1964). Chromosomes were arranged in pairs depending on their FISH signals, length, and other chromosome features as described in our previous work (Pellerin et al. 2019; Youn and Kim 2018).

### Genomic DNA extraction and sequencing of ITS sequences

Genomic DNA of nine *Senna* species was extracted from young leaves by using the cetyltrimethylammonium bromide (CTAB) method (Allen et al. 2006). The ITS primer pairs (F: GTCGCTCCTACCGATTGAA; R: TCTTTTCCTCCGCTTATTGA) were designed based on the 45S rDNA sequence of *S. tora* by using Primer3 software. PCR was performed by initial denaturation at 95 °C for 3 min, followed by 30 cycles of denaturation at 95 °C for 30 s, annealing at 60 °C for 30 s, and extension at 72 °C for 30 s, followed by a final extension at 72 °C for 5 min. Amplicons were ethanol purified and sequenced by Bionics (South Korea).

### Phylogenetic analysis

The *Senna* ITS sequences were aligned using CLC Main Workbench version 5.5, then confirmed through MEGA X. Genetic distance was calculated using the Kimura 2 parameter formula (K2P), and the phylogenetic tree was constructed by MEGA X.

## Results

### Six of the eight ***S. tora*** TRs were detected in the nine ***Senna*** species

**Fig. 1:**
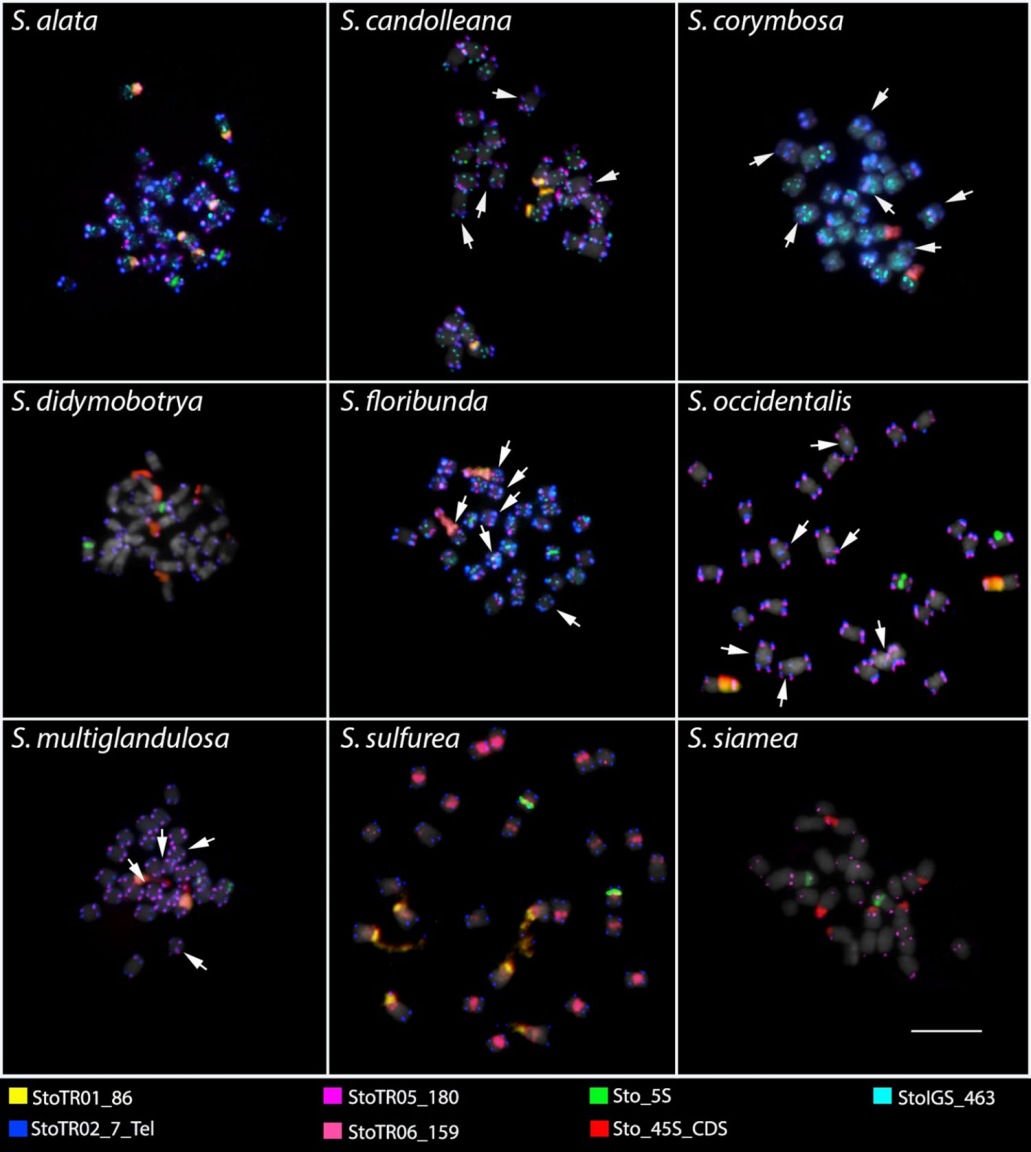
FISH of *S. tora* TRs on root metaphase chromosomes of the nine *Senna* species. Six of the eight TRs, which showed signals from initial FISH screening, and StoIGS_463, are shown here. White arrows indicate the ITRs in *S. candolleana*, *S. corymbosa, S. floribunda, S. occidentalis*, and *S. multiglandulosa*. For signal patterns of individual probes, see supplementary data. Scale bar = 10 µm

All *Senna* species in this study had 2*n* = 28 chromosomes (Fig. 1). In contrast, *S. tora* has only 2*n* = 26 chromosomes (Pellerin et al. 2019). To understand the impact of TRs in the karyotype dysploidy of *S. tora*, we performed FISH to analyze the presence or absence of signals and variations in chromosomal distributions to examine the dynamics of TRs identified in the *S. tora* genome.

Out of the eight *S. tora* TRs (Waminal et al. 2021), two were not detected by FISH in all nine *Senna* species (StoTR03_178 and StoTR04_55), while the other six were either present in all or only a few species (Table 2). StoTR02_7_tel, StoTR05_180, Sto_5S, and Sto_45S_CDS were detected in all species, whereas StoTR01_86 and StoTR06_159 were observed only in eight and four species, respectively (Fig. 1; Table 1). In addition, StoIGS_463 was also observed in only four species.

**Table 2:** Chromosomal distribution of major *Senna tora* tandem repeats in nine *Senna* species

No.	Species	2*n*	StoTR01_86	StoTR02_7_Tel	StoTR05_180	StoTR06_159	Sto_5S	Sto_45S_CDS	StoIGS_463
1	*S. alata*	28	NOR (2S, 7S, 11S)^a^	Tel (all)	sTel (all)	NOR (2S, 7S, 11S)	IR (13S)	NOR (2S, 7S, 11S)^a^	sTel (1–6, 8, 10, 12–14) IRs (1–12, 14)
2	*S. candolleana*	28	NOR (4S, 11S)	Tel (all) IRs (1L, 7L)	sTel (all) IRs (1L, 7L)	–	IR (13S)	NOR (4S, 11S)	sTel (1–3, 5–8, 10, 12–14) IRs (all)
3	*S. corymbosa*	28	NOR (11S)	Tel (all) IRs (1S, 2S, 3S)	sTel (all) IRs (1S, 2S, 3S)	NOR (11S)	IR (13S)	NOR (11S)	sTel (5–10, 12–14) IRs (1–3, 5, 6, 8, 11, 13)
4	*S. didymobotrya*	28	NOR (1S, 2S, 4S, 11S)	Tel (all)	sTel (all)	–	IR (9L)	NOR (1S, 2S, 4S, 11S)	–
5	*S. floribunda*	28	NOR (13S)	Tel (all) IRs (6S, 11S, 13S)	sTel (all) IRs (6S, 11S, 13S)	NOR (13S)	IR (14L)	NOR (13S)	sTel (1–3, 5–14) IRs (2, 4–14)
6	*S. occidentalis*	28	NOR (2S)	Tel (all); IRs (1S, 3S, 4S)	sTel (all) IRs (1S, 3S, 4S)	–	IR (13L)	NOR (2S)	–
7	*S. multiglandulosa*	28	NOR (5S)	Tel (all); IRs (10L, 11L)	sTel (all) IRs (10L, 11L)	NOR (5S)	IR (13S)	NOR (5S)	
8	*S. sulfurea*	28	NOR (2S, 3S, 7S)	Tel (all)	pCen (all)		IRs (13S)	NOR (2S, 3S, 7S)	–
9	*S. siamea*	28	–	Tel (all)	sTel (all)		IRs (4S)	NOR (2S, 5S, 6S)	–

*NOR* nucleolar organizer region (45S rDNA locus),* Tel* telomeric region,* sTel* subtelomeric region,* IR* interstitial region,* pCen* pericentromere,* S* short arm,* L* long arm

^a^Chromosomal niche occupied by corresponding tandem repeats

^b^Numbers in parenthesis indicate chromosome number with FISH signals for corresponding repeats

### StoTR02_7_Tel and StoTR05_180 were mostly colocalized

The StoTR02_7_Tel, which is an *Arabidopsis*-type telomere repeat sequence, was highly amplified in the interstitial regions of *S. tora* chromosomes in addition to the canonical sites at chromosome termini (Pellerin et al. 2019; Waminal et al. 2021). Likewise, StoTR05_180 was also mostly colocalized with StoTR02_7_Tel in *S. tora* in pericentromeric regions but absent in subtelomeric sites.

**Fig. 2:**
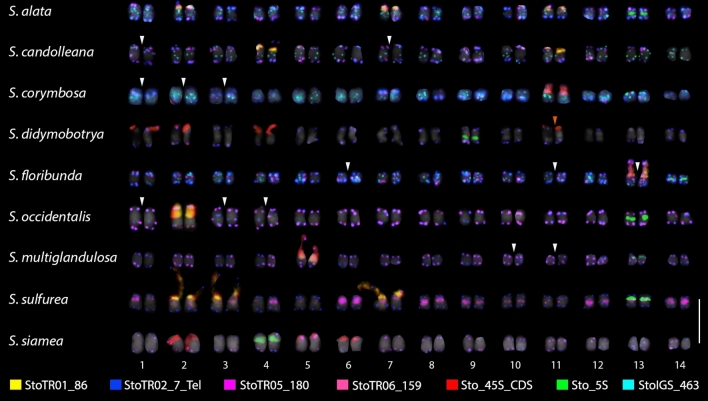
FISH karyograms of the root metaphase chromosomes of the nine *Senna* species. StoTR01_86 was mostly colocalized at 45S rDNA sites in all species except *S. siamea*. StoTR05_180 was at subtelomeric sites in all species except in *S. sulfurea*, where it was localized at pericentromeric regions. The white arrows indicate the ITR in *S. candolleana*, *S. corymbosa, S. floribunda, S. occidentalis*, and *S. multiglandulosa*. The orange arrow shows the weak signal of StoTR01_86 in *S. didymobotrya.* For karyogram of each species showing individual TR distribution, see supplementary data. Scale bar = 10 µm

**Fig. 3:**
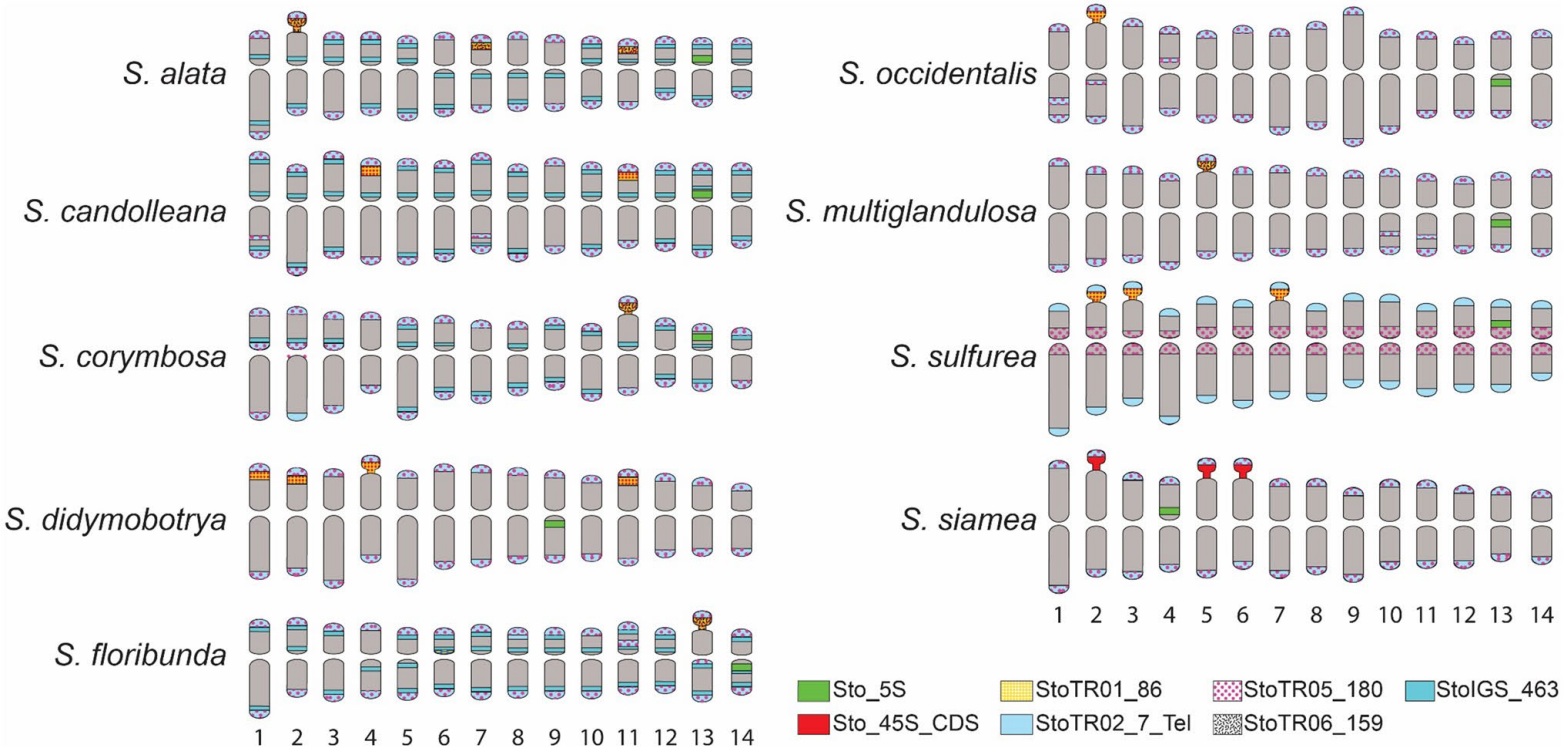
Karyotype idiograms showing the distribution of *S. tora* TRs in nine *Senna* species. Note the colocalization of StoTR01_86 and StoTR06_159 at the 45S rDNA loci in some species, and the unique distribution of StoTR05_180 at the pericentromeric regions of all chromosomes in *S. sulfurea*, contrary to subtelomeric location in other species

**Fig. 4:**
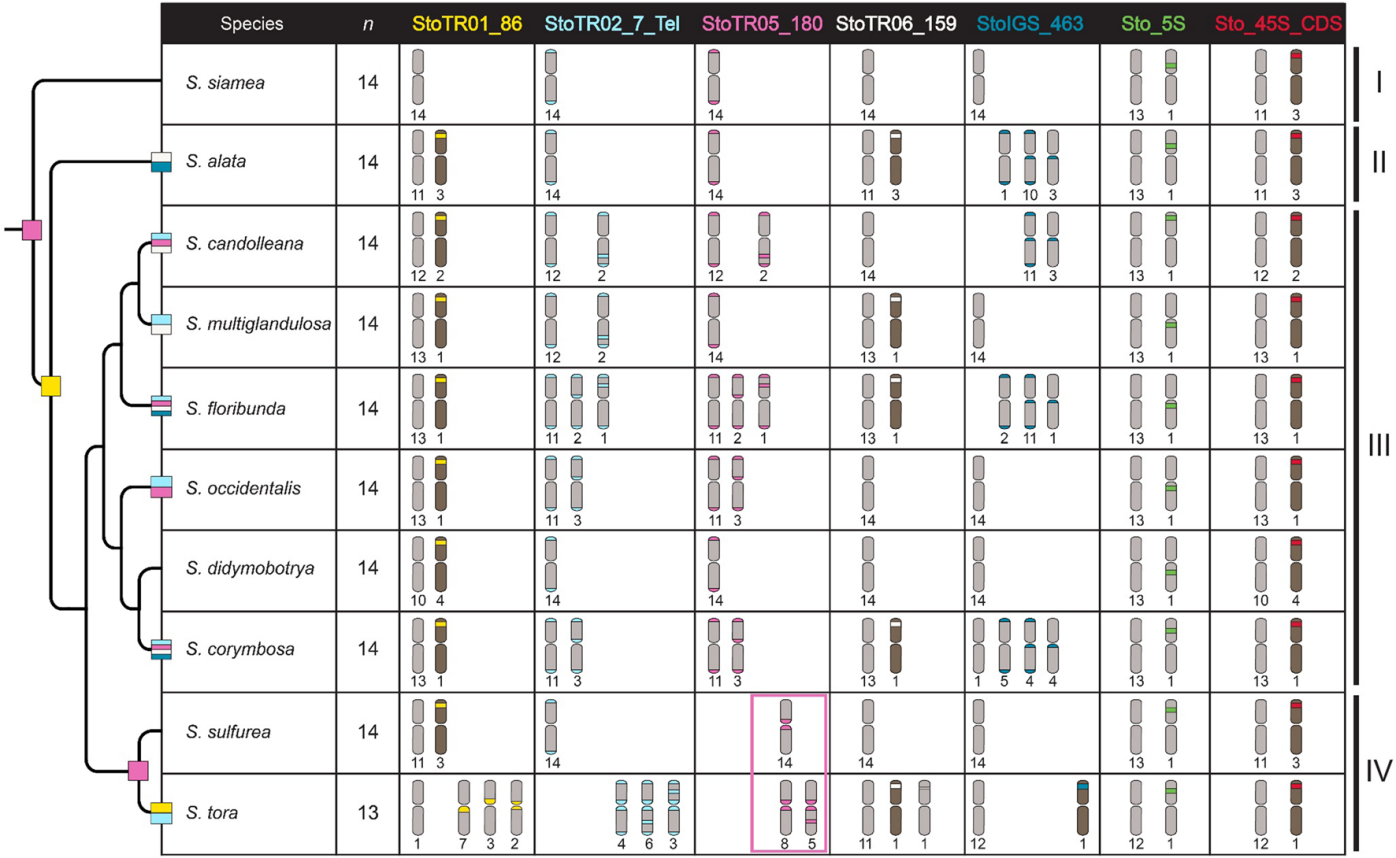
Cyto-phylogenetic analysis among *Senna* species. The ten species, including *S. tora*, were grouped into four (I–IV). StoTR05_180 at the subtelomeric site is shared in all ten, and likely the primitive distribution pattern in, *Senna*. StoTR01_86 was shared by species in Groups II–IV. Independent chromosomal rearrangements involving several repeats and chromosomes have taken place in species Groups II–IV. Species in Group IV shared rearrangements involving the displacement of StoTR05_180 to the pericentromeric regions of chromosomes (pink box)

In the present study, all nine *Senna* species had StoTR02_7_Tel signals at the terminal region of all chromosomes (Fig. 1). However, in addition to these canonical sites, interstitial telomeric repeat (ITR) signals were also detected in *S. candolleana*, *S. corymbosa*, *S. floribunda*, *S. multiglandulosa*, and *S. occidentalis* (Figs. 2, 3 and 4; Table 2). In *S. candolleana*, chromosomes 1 and 7 had ITRs in the long arm. In *S. corymbosa*, ITRs were detected in the proximal region of the short arm of chromosomes 1, 2, and 3. In *S. floribunda*, three chromosomes had ITRs in the short arm; those in chromosomes 6 and 13 were in proximal regions while those in chromosome 11 were more interstitial. In *S. occidentalis*, ITRs were located at proximal regions in the short arm of chromosomes 1, 3, and 4.

Meanwhile, StoTR05_180 signals were detected as colocalized signals with StoTR02_7_Tel in all species, except in *S. sulfurea* (Figs. 2, 3 and 4). In *S. sulfurea*, StoTR05_180 did not colocalize with StoTR02_7_Tel. StoTR02_7_Tel was exclusively distributed at chromosome termini in *S. sulfurea*, whereas StoTR05_180 was absent in these regions but was amplified in the pericentromeric regions with varied intensities in all chromosomes (Fig. 2).

### The number of 45S rDNA loci was more diverse than that of 5S rDNA

Similar to *S. tora*, all nine species had only one pair of Sto_5S rDNA, whereas Sto_45S rDNA varied from one to four loci (Table 1). The 5S rDNA signals were localized in the short arms of the respective chromosomes in *S. alata*, *S. candolleana*, *S. corymbosa*, *S. multiglandulosa*, *S. sulfurea*, and *S. siamea*, and in the long arms in *S. didymobotrya*, *S. floribunda*, and *S. occidentalis* (Figs. 2, 3; Table 2).

**Fig. 5:**
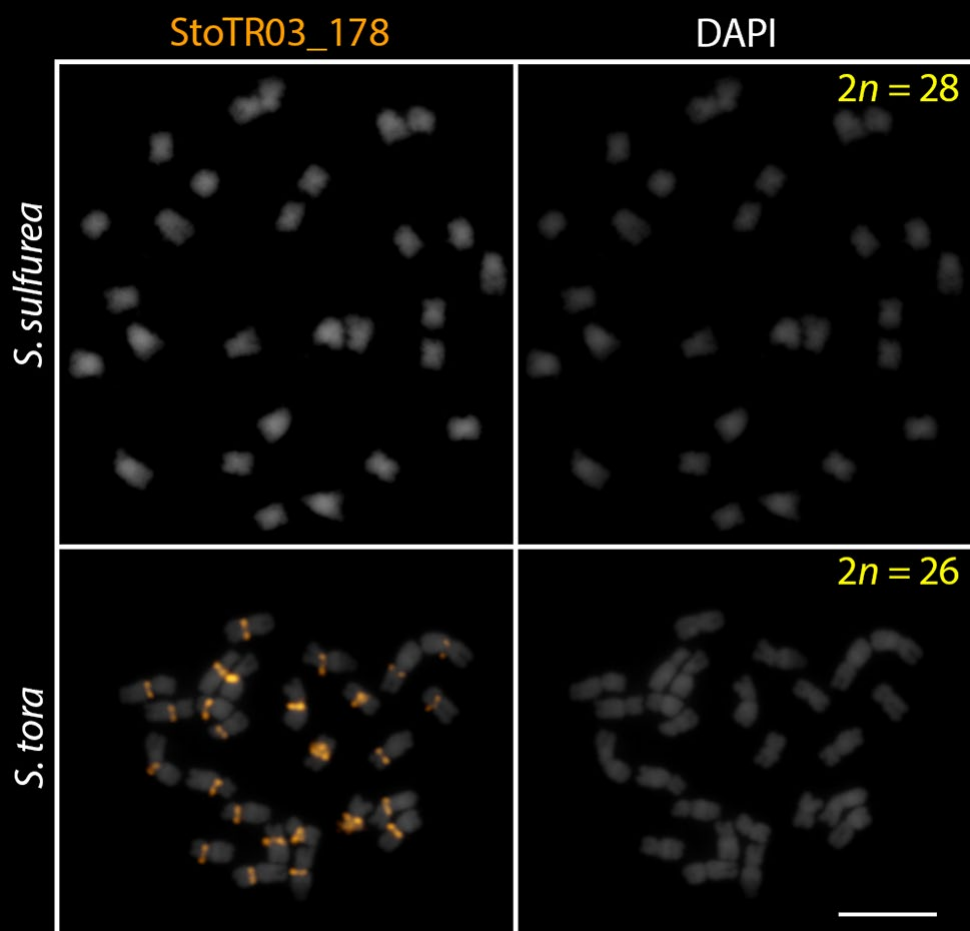
Comparative FISH with StoTR03_178 between *S. sulfurea* and *S. tora.* FISH signals were only detected in *S. tora*. Scale bar = 10 µm

The Sto_45S_CDS signals were distributed only in the subtelomeric regions of the short arms of the respective chromosomes. One pair of 45S rDNA was detected in *S. corymbosa, S. floribunda*, *S. multiglandulosa*, and *S. occidentalis*, two in *S. candolleana*, three in *S. alata*, *S. siamea*, and *S. sulfurea*, and four in *S. didymobotrya* (Figs. 2, 5; Table 2).

### StoTR01_86 and StoTR06_159 colocalized at the 45S rDNA loci

The chromosomal distribution of StoTR01_86 in *S. tora* was in total contrast with that observed in the *Senna* species in this study. Although StoTR01_86 was localized in the pericentromeric regions in all *S. tora* chromosomes (Waminal et al. 2021), it was absent in the pericentromeric region in all currently investigated *Senna* species but colocalized at all 45S rDNA loci, except in *S. siamea*, which did not show any StoTR01_86 signal at all (Figs. 2, 3 and 4; Table 2).

On the other hand, StoTR06_159 was colocalized with 45S rDNA in *S. tora*, and also in *S. alata*, *S. corymbosa*, *S. floribunda*, and *S. multiglandulosa* (Figs. 2, 3 and 4; Table 2). However, the minor extra-NOR StoTR06_159 locus observed in *S. tora* was not detected in species with StoTR06_159 signals. None of the other species showed any StoTR06_159 signals (Table 2).

### StoIGS_463 localized at extra-NOR loci

StoIGS_463 is a 463 bp duplicated sequence with two copies identified in the IGS region of *S. tora* 45S rDNA (Waminal et al. 2021). FISH also confirmed the exclusive localization of StoIGS_463 at the NOR site in *S. tora*. However, FISH on the nine *Senna* species showed no signals at NOR sites, but rather at subtelomeric or interstitial chromosomal regions in some species and were completely absent in some species (Figs. 2, 3; Table 2). While some distinct paired signals were observed, dispersed signals were also detected in many chromosomes, such as those in *S. corymbosa*, indicative of transposable element FISH signals.

Of the nine species, only *S. alata, S. candolleana, S. corymbosa*, and *S. floribunda* showed StoIGS_463 FISH signals (Figs. 2, 3 and 4; Table 2). Aside from chromosomes without signals, three StoIGS_463 distribution patterns were observed in these four species: (1) at the subtelomeric and interstitial regions, (2) only in the subtelomeric regions, and (3) only in the interstitial regions. In *S. alata*, there were ten, one, and three chromosomes that had the first, second, and third StoIGS_463 distribution patterns, respectively (Fig. 4; Table 2). In *S. candolleana*, only the first and third patterns were observed with eleven and three chromosomes bearing each pattern, respectively (Fig. 4; Table 2). In *S. corymbosa*, chromosome 4 did not show any StoIGS_463 signal, while four, five, and four chromosomes showed patterns i, ii and iii, respectively (Fig. 4; Table 2). Finally, in *S. floribunda*, there were 11, two, and one chromosomes that showed patterns i, ii and iii, respectively (Fig. 4; Table 2).

### Chromosome rearrangement patterns support ITS-based phylogenetic tree

Since many TR loci formed from chromosomal rearrangements are not readily visible by FISH when these loci are shorter than the FISH detection threshold, grouping species based solely on FISH signal patterns could be misleading. Therefore, we compared the FISH distribution with the phylogenetic tree inferred using the entire ITS sequences (ITS1-5.8S-ITS2) of the *Senna* species, including *S. tora* data from our previous work (Waminal et al. 2021).

The *Senna* ITS length ranged from 632 bp in *S. tora* to 663 bp in *S. corymbosa* and had a mean of 649 bp. The ITS1 and ITS2 had relatively higher GC contents than those of 5.8S, and the GC content of the entire sequence ranged from 58.54–62.84 % and averaged 61.45 % (Table 3).

**Table 3:** Sequence length and GC content of the 45S rDNA ITS1-5.8S-ITS2 sequences of the 10 *Senna* species

No.	Sample	ITS1		5.8S		ITS2		Total (bp)	
		bp	GC%	bp	GC%	bp	GC%	bp	GC%
1	*S. alata*	252	64.68	158	53.8	237	63.71	647	61.67
2	*S. candolleana*	257	63.81	160	54.37	237	67.51	654	62.84
3	*S. corymbosa*	256	62.11	160	54.37	247	65.59	663	61.54
4	*S. didymobotrya*	251	62.15	158	55.41	237	65.13	646	61.61
5	*S. floribunda*	250	63.60	160	54.37	235	67.23	645	62.64
6	*S. occidentalis*	256	60.16	160	54.37	236	64.83	652	60.43
7	*S. multiglandulosa*	257	63.04	160	54.37	237	66.67	654	62.23
8	*S. tora*	236	61.86	160	53.75	236	58.47	632	58.54
9	*S. siamea*	255	63.92	158	53.8	235	62.55	648	60.96
10	*S. sulfurea*	256	64.45	160	53.75	237	64.98	653	62.02
Mean		253	62.98	159	54.24	237	64.67	649	61.45

These ITS sequences divided the *Senna* species into four (Fig. 4). Group I comprised only *S. siamea*, which showed the fewest FISH signals of the *S. tora* TRs, and the most primitive chromosomal distribution pattern, StoTR05_180, at the subtelomeric regions of all chromosomes. Groups II–IV all had StoTR01_86 signals, mostly at the 45S rDNA loci. Independent chromosomal rearrangements involving the other repeats have taken place in the species in these groups. The absence of FISH signals may either be short arrays that make these loci undetectable using FISH or indicate a lack of actual rearrangements. Additional chromosomal rearrangements involving StoTR05_180 occurred in Group IV. These rearrangements displaced the subtelomeric location of StoTR05_180 into the pericentromeric region, where they have been highly amplified in both *S. sulfurea* and *S. tora*. Additional rounds of rearrangements may have occurred in *S. tora*, as shown by several chromosomes with interstitial StoTR05_180 signals.

## Discussion

In our study, we have compared the chromosomal distribution of the TRs identified from the *S. tora* genome with nine other *Senna* species in order to understand the impacts and evolutionary dynamics of these TRs in the dysploidization of the *S. tora* karyotype. Compared with the nine *Senna* species assessed in this study, *S. tora* had the most extensive chromosomal rearrangements and the only one with a descending dysploidy karyotype (Fig. 4), suggesting the involvement of these TRs in shaping the extant *S. tora* genome.

### Interstitial telomeric repeats are evidence for ***Senna*** chromosomal rearrangements

In most eukaryotes, telomeric repeats are usually located at the terminal end of chromosomes and have a key function in preventing chromosomal damage (Muraki et al. 2012). However, telomeric repeats have also been found in interstitial regions, also known as interstitial telomeric repeats (ITRs) (Bolzan 2012). Several mechanisms have been postulated to explain the formation of ITRs. These include interchromosomal telomere fusion, DNA polymerase slippage, and double-strand break repair (Uchida et al. 2002; Lin and Yan 2008).

Of the ten *Senna* species, including *S. tora*, six had readily detectable ITR signals indicating shared chromosomal rearrangements involving StoTR02_7_Tel among these *Senna* species. The lack of ITR signals in the other species may indicate fixation and reduction of these repeat loci, rendering them undetectable by FISH, as has been previously observed in other plants (He et al. 2013). It is also possible that these StoTR02_7_Tel-mediated chromosomal rearrangements occurred independently in each species. The extreme contrast of the StoTR02_7_Tel distribution between *S. siamea* and *S. tora* suggests that *S. siamea* has a more primitive karyotype and that the massive chromosomal rearrangements in *S. tora* may have evolved relatively recently.

### Concerted conversion of StoTR05_180 to (peri)centromeric TRs in ***S. sulfurea*** and ***S. tora***

Species in Groups I–III carried StoTR05_180 in the subtelomeric regions, whereas those in Group IV (*S. sulfurea* and *S. tora*) have somehow managed to transpose these TR loci from the subtelomeric to the pericentromeric region in all chromosomes. We proposed the hypothesized that this transposition has been occurred involving all chromosomes of the group IV ancestral karyotype. As a result, the evidence indicated that StoTR05_180 presents in the pericentromeric region of all chromosomes in *S. sulfurea* and *S. tora.* The other scenario is likely given the relatively random process of chromosomal rearrangement events.

One possible mechanism for this concerted TR array transposition may involve chromoplexy, which is a massive chromosomal rearrangement event that involves several chromosomes (Comai and Tan 2019; Pellestor and Gatinois 2020). Microhomologies between telomeric and (peri)centromeric regions make these regions hotspots for chromosomal inversions (He et al. 2013).

While both *S. sulfurea* and *S. tora* had lost subtelomeric StoTR05_180 loci but amplified pericentromeric loci, only *S. tora* developed a novel centromeric repeat, StoTR03_178 (Fig. 5), which may have likely evolved from StoTR05_180 (Waminal et al. 2021). Disruption of the epigenetic makeup immediately after chromosomal rearrangements may have enabled StoTR05_180 to function as a novel centromeric repeat. This, in *S. tora*, may have eventually caused centromere positioning, and fixation of StoTR03_178 variants.

### The 45S rDNA IGS as repeat carrier during chromosomal rearrangements

45S rDNA is associated with genome rearrangements (Havlová et al. 2016). The sub-repeat elements in the IGS are considered important players in the dynamics of IGS (Jo et al. 2011). The elimination and reorganization of the IGS repeat elements were observed in *Nicotiana tabacum* after allopolyploidization which caused IGS length variation among *Nicotiana* species (Volkov et al. 1999). Although there is no exact mechanism to clearly explain the movement of TRs in and out of the 45S rDNA IGS, some authors have observed that TRs in the IGS moved out and amplified in another chromosomal region (Almeida et al. 2012). However, others also noted the opposite direction of IGS TR evolution in *Phaseolus vulgaris*, such that TRs moved into the 45S rDNA IGS from another region in the genome (Falquet et al. 1997). Our data suggest that the 45S rDNA IGS can act as TR “carrier” during chromosomal rearrangements, and that fragments of transposed TRs could sometimes get lodged and fixed in a taxon. Moreover, this observation suggests that TRs in the IGS are more like “footprints” of the recent involvement of the IGS in chromosomal rearrangements.

StoTR01_86, which was at the 45S rDNA locus in most *Senna* species except *S. siamea* and *S. tora*, suggests that 45S rDNA is involved in chromosomal rearrangement after the divergence of the *Senna* species from the ancestral karyotype *S. siamea*, which resulted in the fixation of StoTR01_86 in Group II-III species. Eventually, chromosomal rearrangement specific to *S. tora* may have caused more recent expulsion of the StoTR01_86 from the IGS and moved it into the pericentromeric regions of all chromosomes (Fig. 4) (Waminal et al. 2021).

StoTR06_159 also illustrates the movement of TRs in and out of the 45S rDNA IGS (Fig. 4). Moreover, StoIGS_463 explicitly showed the active role of the 45S rDNA IGS in homing repeats, which in this case is the subtelomeric ancestral repeat of StoIGS_463.

### FISH data suggest the involvement of TRs in ***S. tora*** dysploidy

In *Senna*, the basic chromosome number is *x* = 14 (Waminal et al. 2021). Several species, however, have *x* = 11, 12, 13 resulting from descending dysploidization events (Elaine et al. 2005). Although various *Senna* species showed FISH signals from *S. tora* TRs, only *S. tora* had extensive genome rearrangements and dysploid karyotype (Fig. 4), suggesting the involvement of TRs in *S. tora* karyotype dysploidy.

Moreover, StoTR03_178 was observed in the interstitial region of *S. tora* chromosome 7, suggesting a relatively recent chromosome fusion (Waminal et al. 2021). Although we are not yet certain which ancestral chromosomes fused to form the *S. tora* chromosome 7, it is likely that orthologous *S. sulfurea* chromosomes 1 and 4 may have been involved. This hypothesis is based on the presence of subtelocentric *S. sulfurea* chromosomes 1 and 4, which were not detected in *S. tora* (Fig. 3). Moreover, subtelocentric chromosomes are often involved in interchromosomal fusion (Schubert and Lysak 2011).

## Conclusions

We compared the chromosomal distribution of the eight *S. tora* TRs and a 45S rDNA IGS duplicated sequence, StoIGS_463, among nine *Senna* species. We have seen the dynamics of these TRs, which showed both shared and independent evolution. Importantly, we have shown cytogenetic evidence of the bidirectionality of TR movement into or out of the 45S rDNA IGS region, suggesting a role for 45S rDNA as a repeat carrier during chromosomal rearrangements. Moreover, these data also provide cytogenetic visualization of the expansion, contraction, and reorganization of repeat families in a repeat “library” of a lineage. Further studies should focus on characterizing the 45S rDNA IGS sequences of all *Senna* species to gain more insight into the role of the IGS in genome rearrangements.

## Electronic Supplementary Material

Below is the link to the electronic supplementary material.

Supplementary Material 1
