# Supplementary material for: Comparative FISH analysis of Senna tora tandem repeats revealed insights into the chromosome dynamics in Senna
Source: Genes Genomics. 2021 Mar 3;43(3):237–49. doi: 10.1007/s13258-021-01051-w (PMC7966213; doi:10.1007/s13258-021-01051-w)
Supplement: Supplementary file 1 — Supplementary Material 1 [file 13258_2021_1051_MOESM1_ESM.docx]

**Supplementary Information**


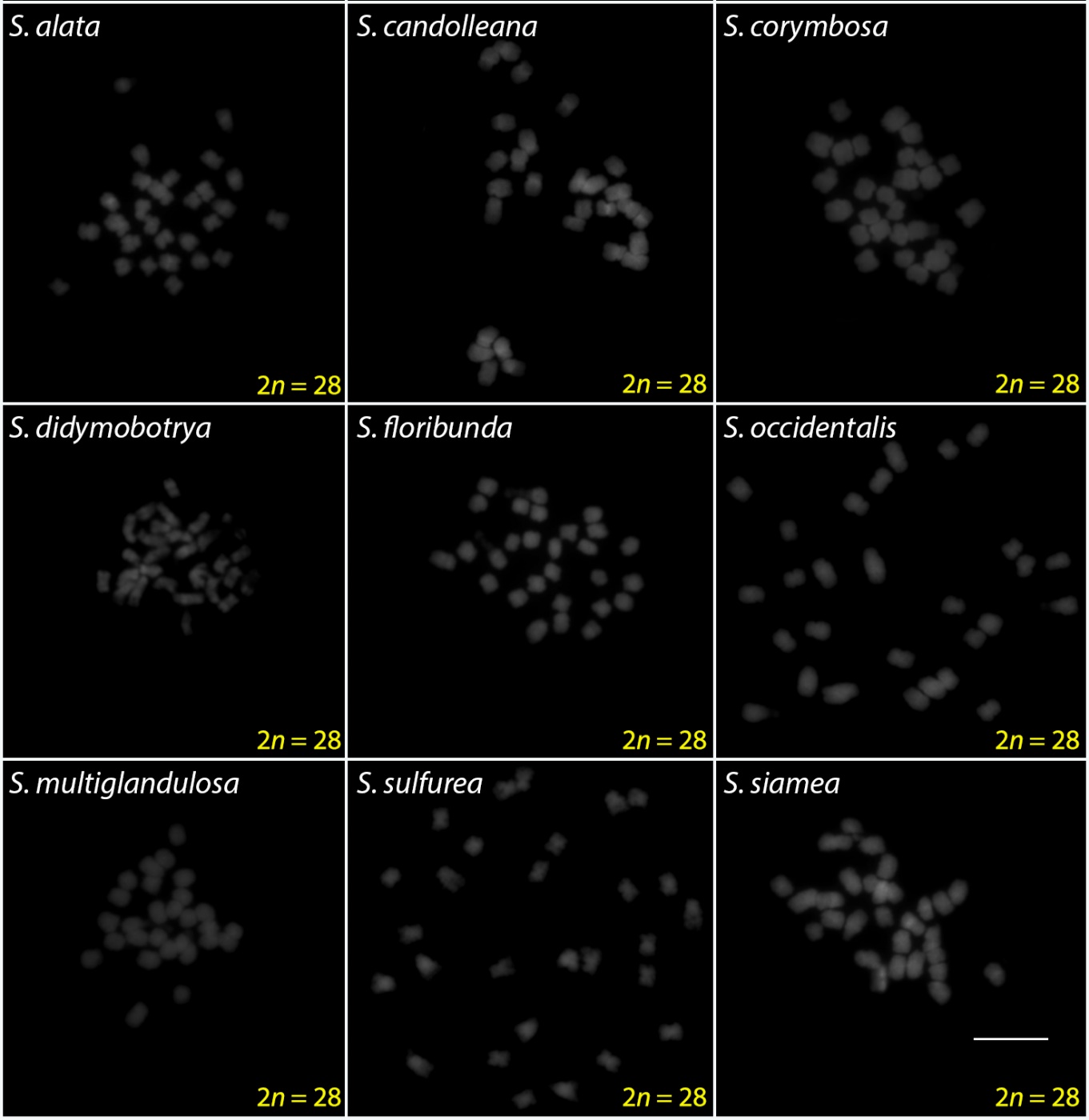


**Fig. S1** Metaphase spreads of nine *Senna* species. Scale bar = 10 µm


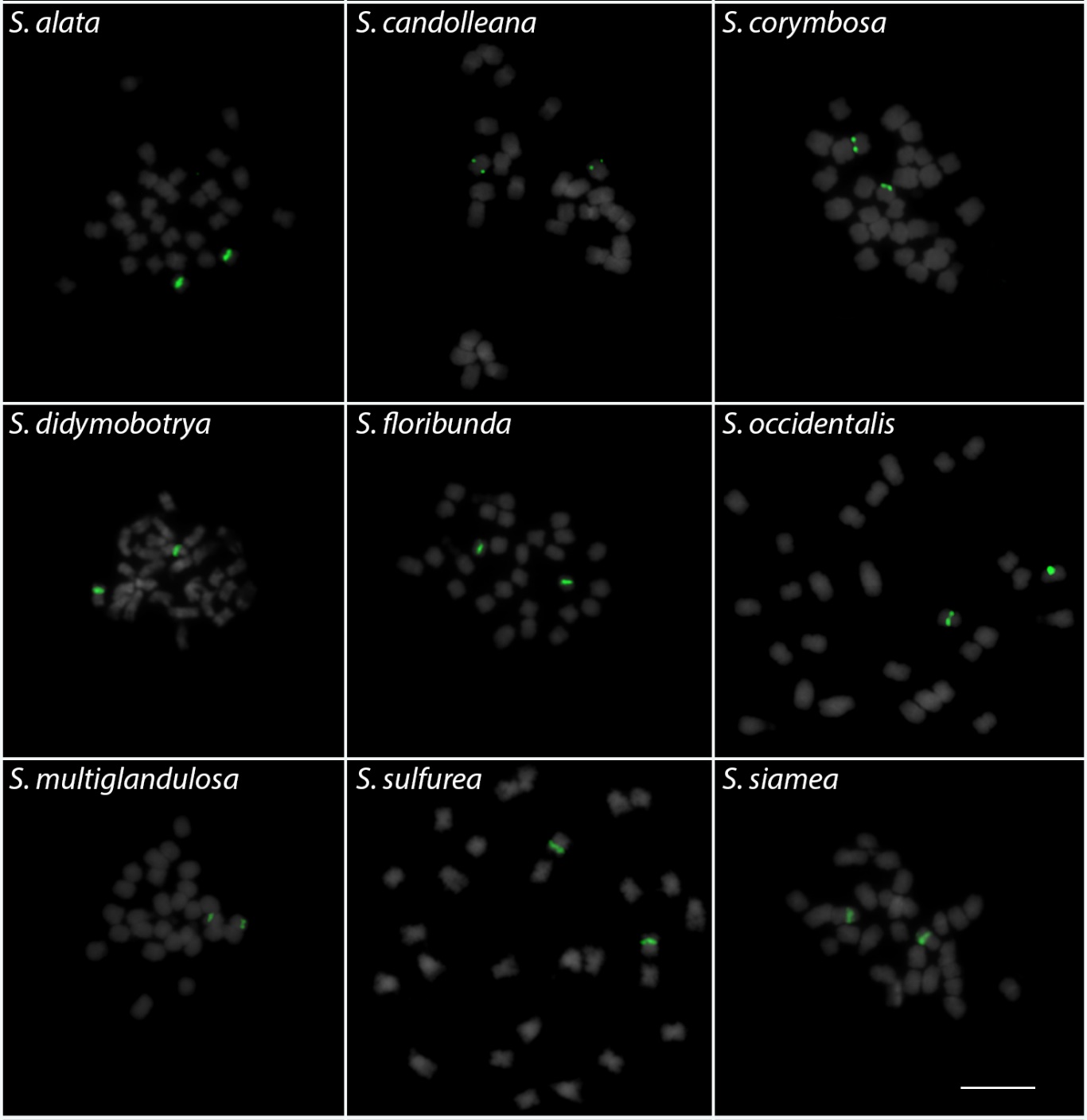


**Fig. S2** Distribution of Sto_5S on the metaphase spreads of nine *Senna* species. The Sto_5S signals are labeled by green. Scale bar = 10 µm


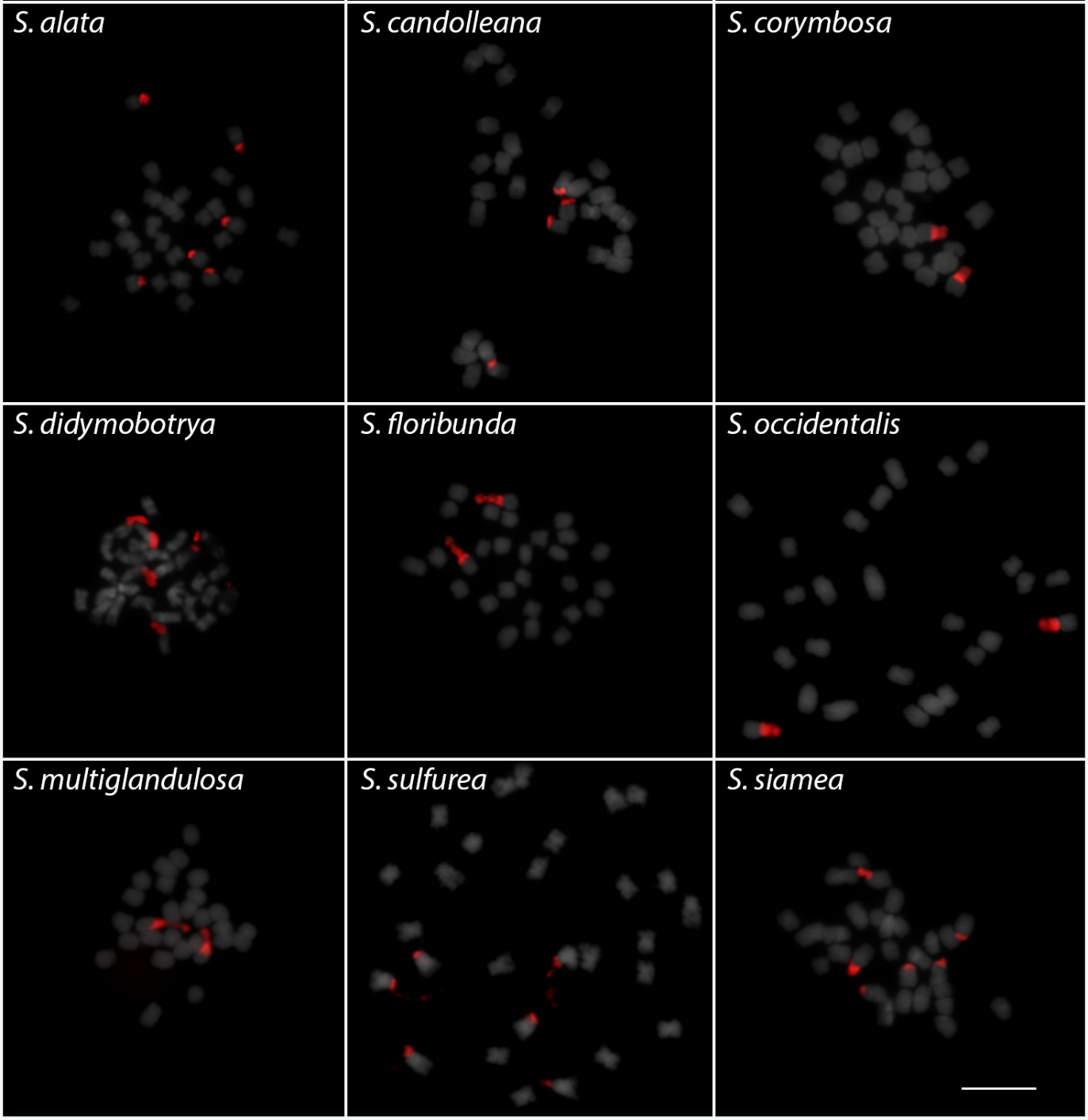


**Fig. S3** Distribution of Sto_45S_CDS on the metaphase spreads of nine *Senna* species. The Sto_45S_CDS signals are labeled by red. Scale bar = 10 µm


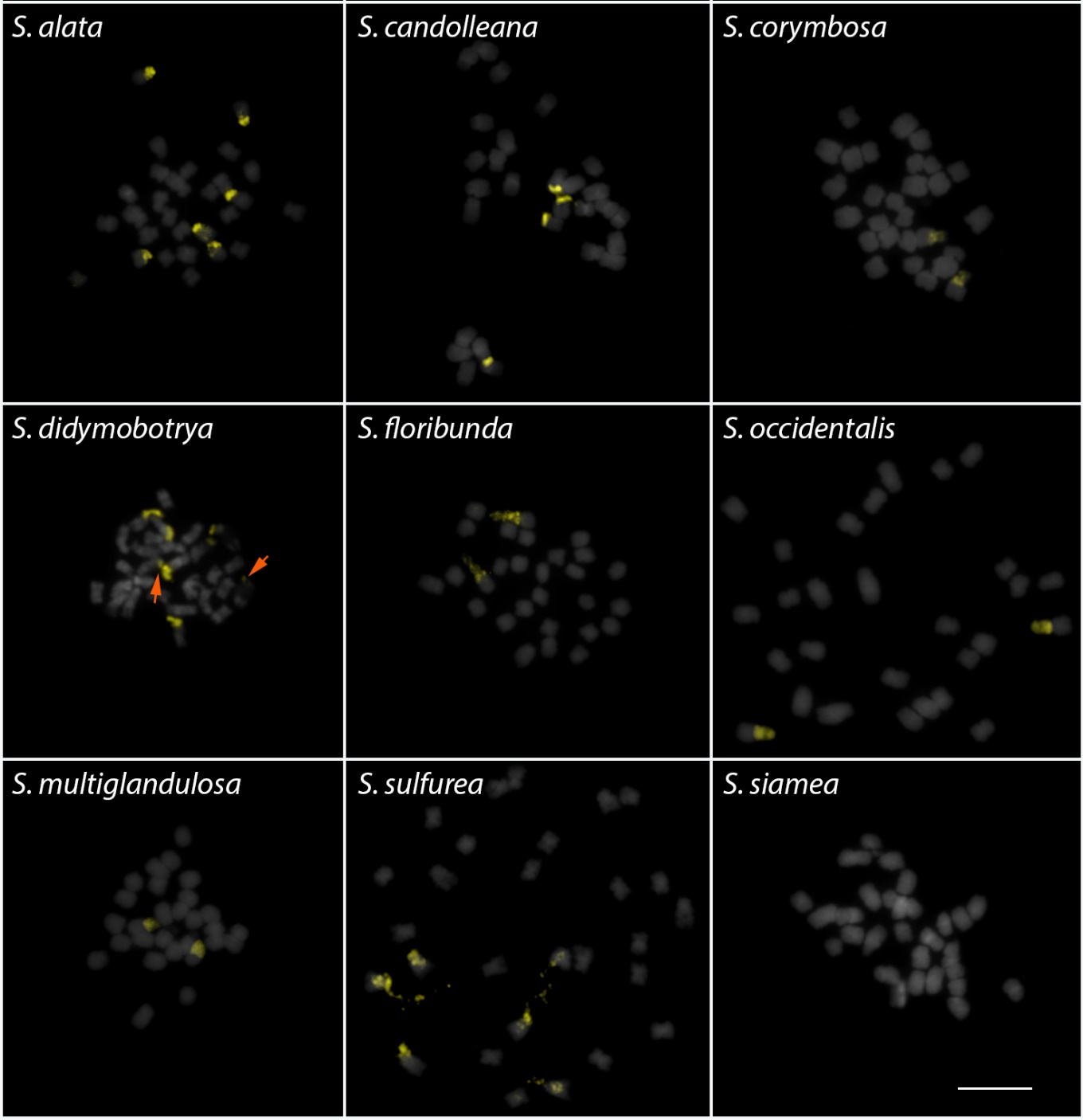


**Fig. S4** Distribution of StoTR01_86 on the metaphase spreads of nine *Senna* species. The StoTR01_86 signals are labeled by yellow. The yellow arrows show the weak signal of StoTR01_86 in *S. didymobotrya.* Scale bar = 10 µm


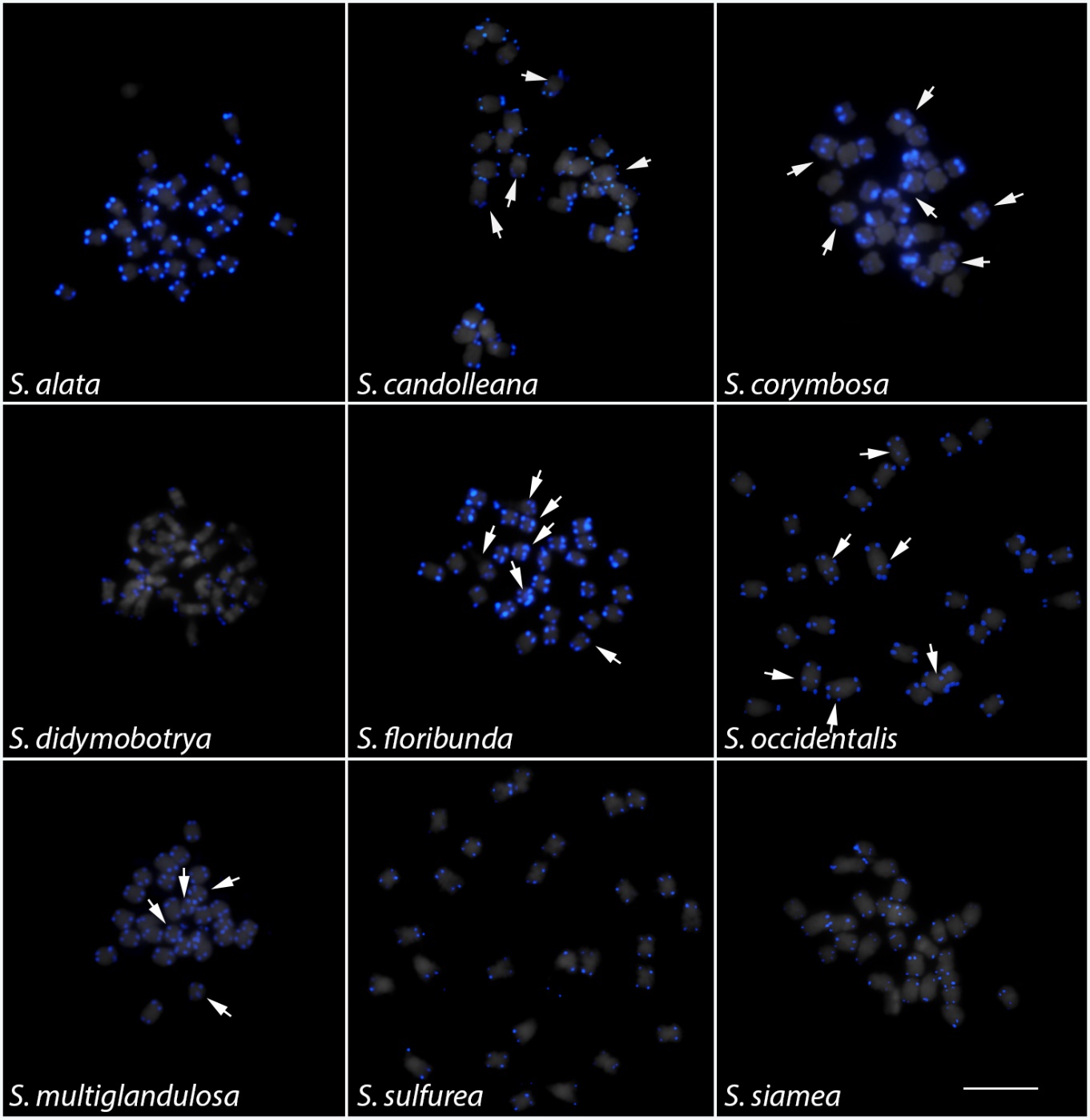


**Fig. S5** Distribution of StoTR02_Tel_7 on the metaphase spreads of nine *Senna* species. The white arrows indicate the ITR signals on the chromosomes. The StoTR02_Tel_7 signals are labeled by blue. Scale bar = 10 µm


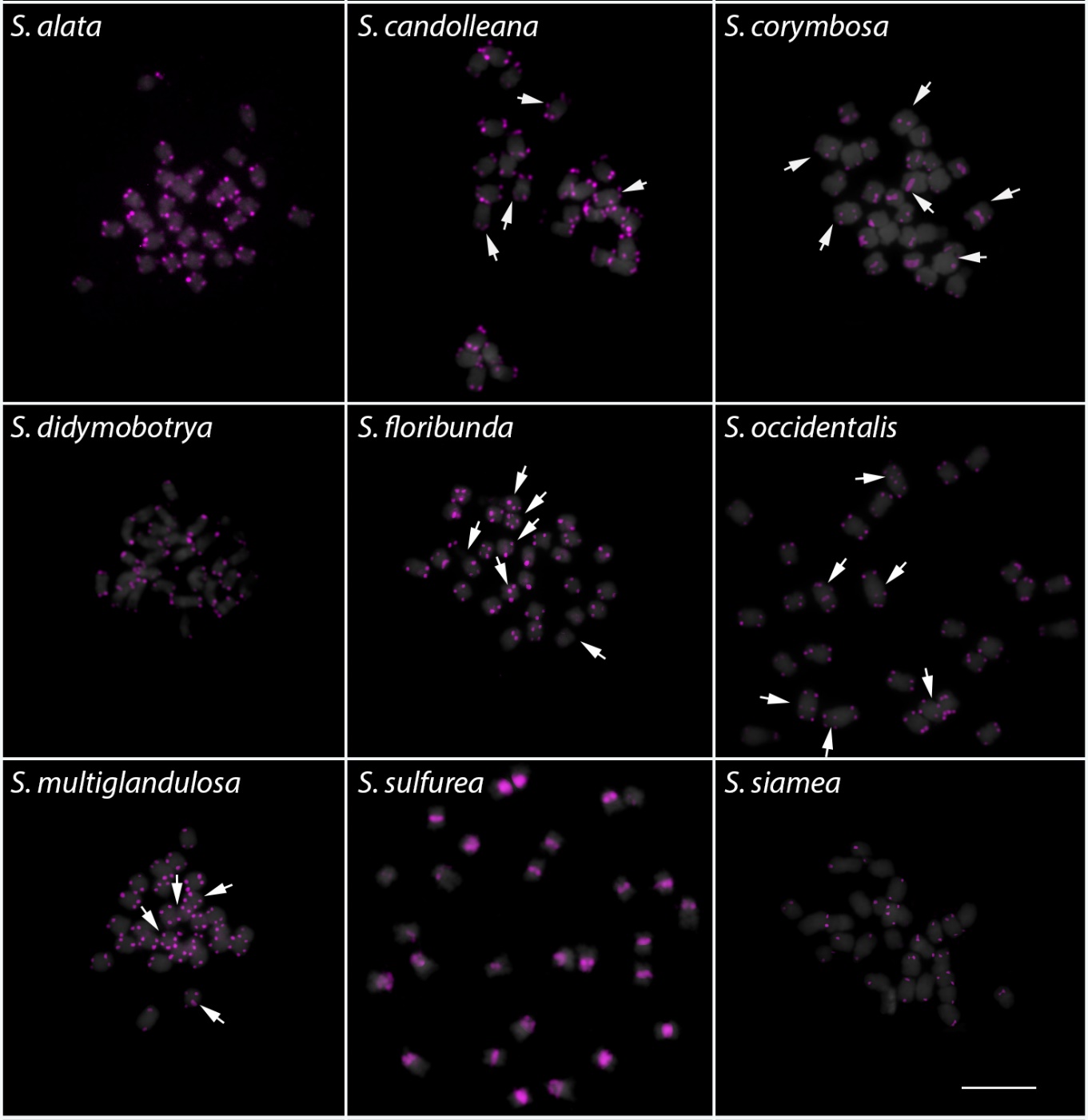


**Fig. S6** Distribution of StoTR05_180 on the metaphase spreads of nine *Senna* species. The white arrows indicate the ITR signals on the chromosomes. The StoTR05_180 signals are labeled by violet. Scale bar = 10 µm


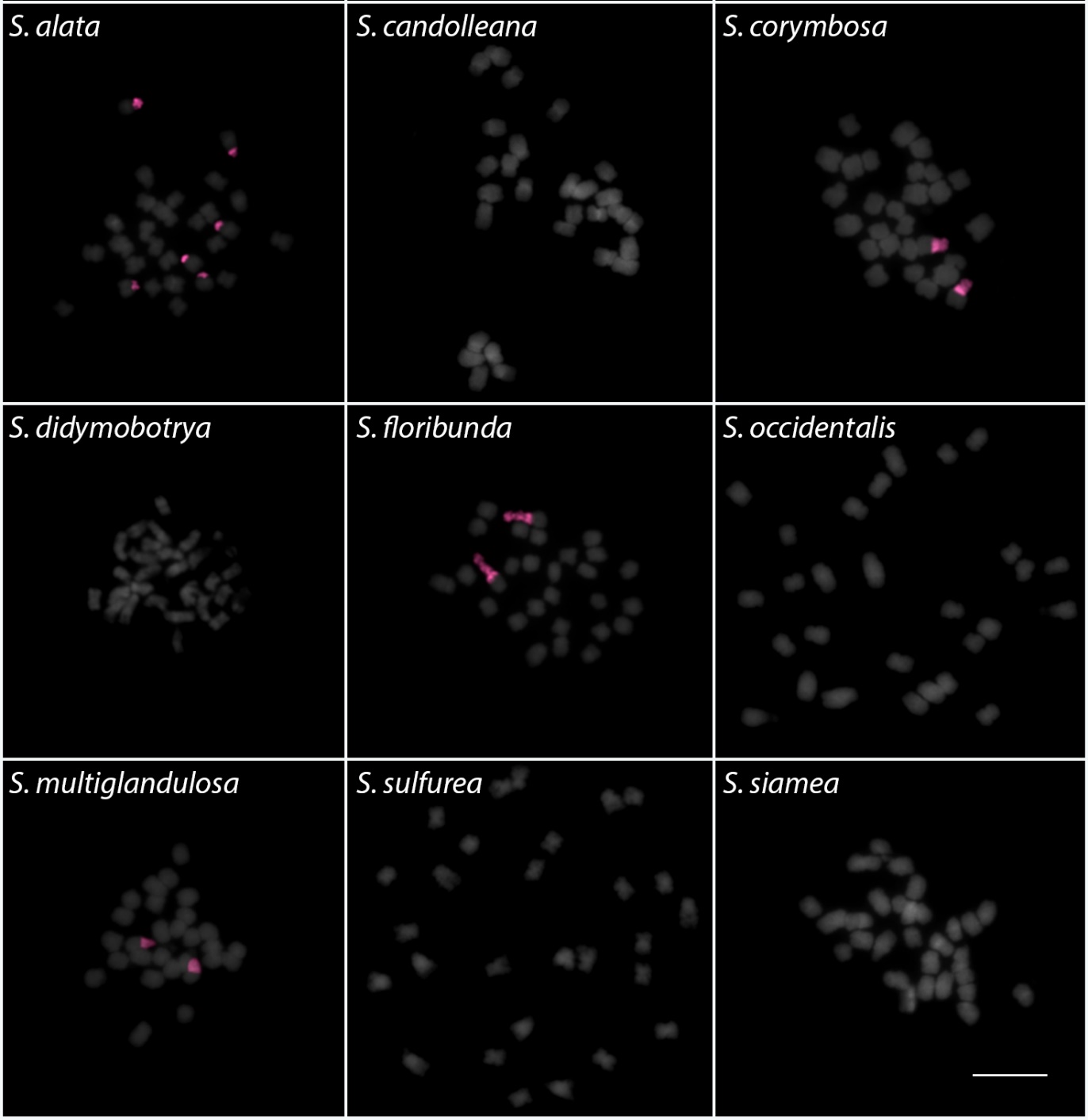


**Fig. S7** Distribution of StoTR06_159 on the metaphase spreads of nine *Senna* species. The StoTR06_159 signals are labeled by pink. Scale bar = 10 µm


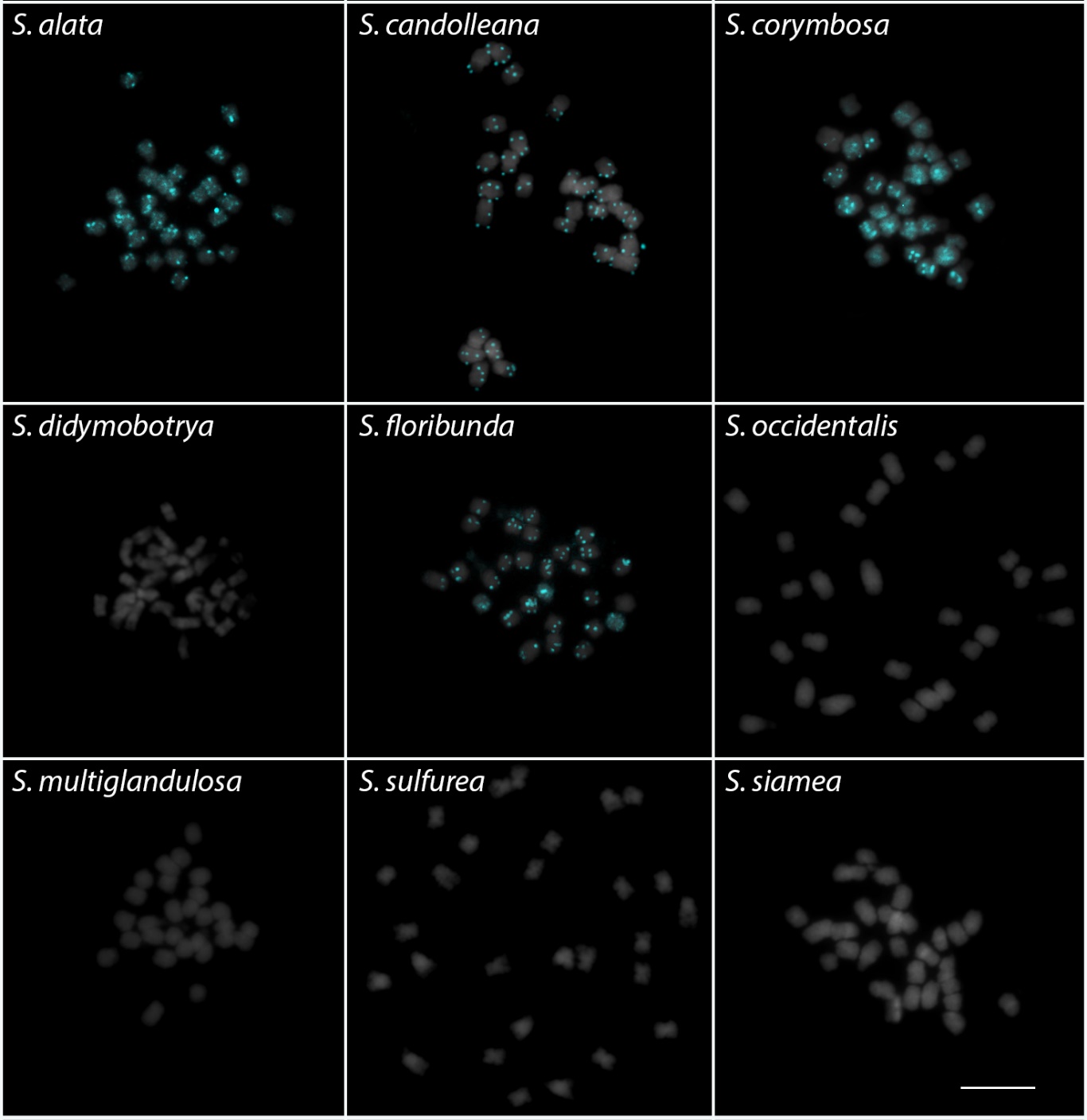


**Fig. S8** Distribution of StoIGS_463 on the metaphase spreads of nine *Senna* species. The StoIGS_463 signals are labeled by cyan. Scale bar = 10 µm


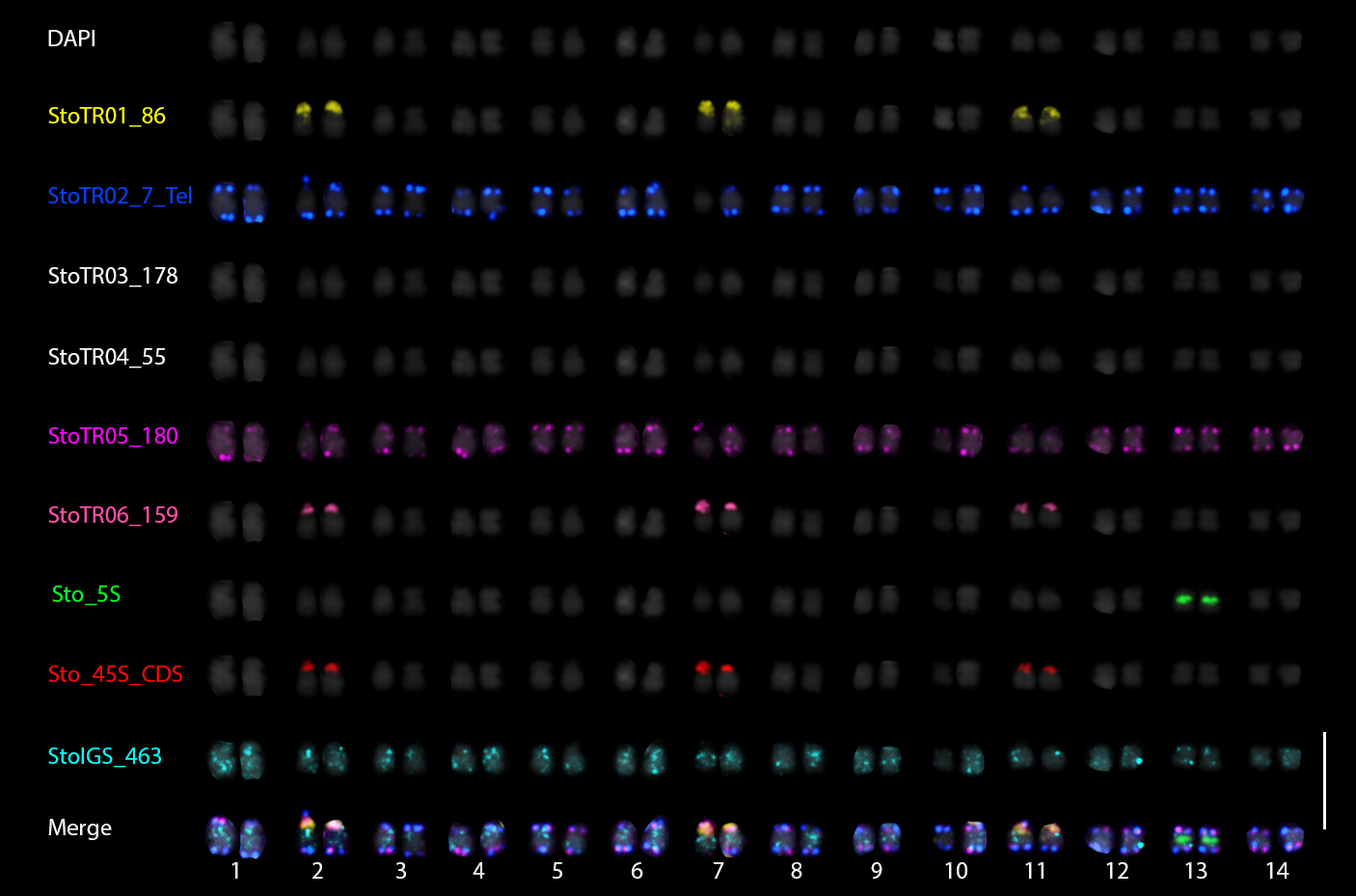


**Fig. S9** FISH karyograms of *S. alata* with nine TRs probes. Scale bar = 10 µm


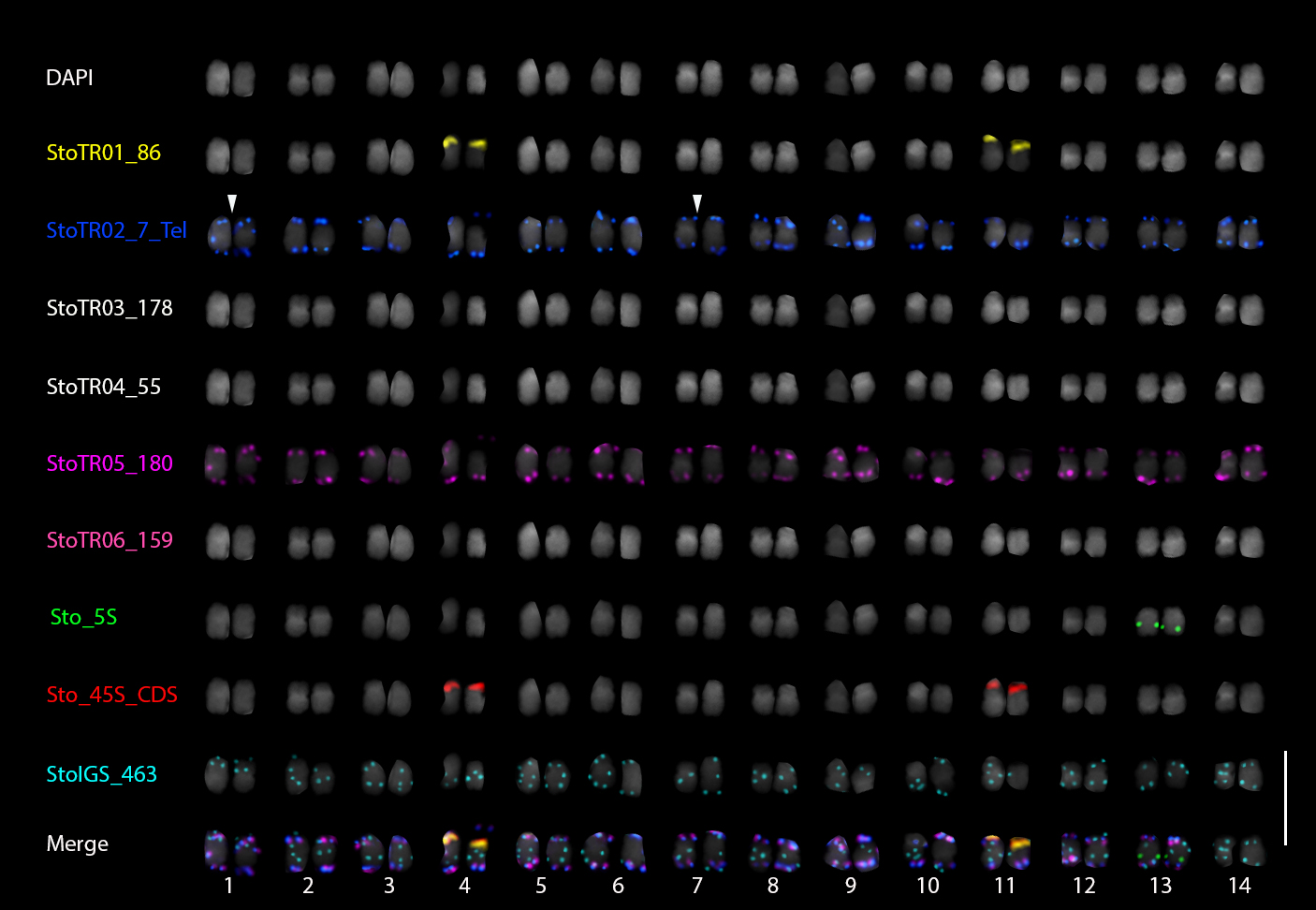


**Fig. S10** FISH karyograms of *S. candolleana* with nine TRs probes. The white arrows show the ITR signals on the chromosomes 1, and 7. Scale bar = 10 µm

**
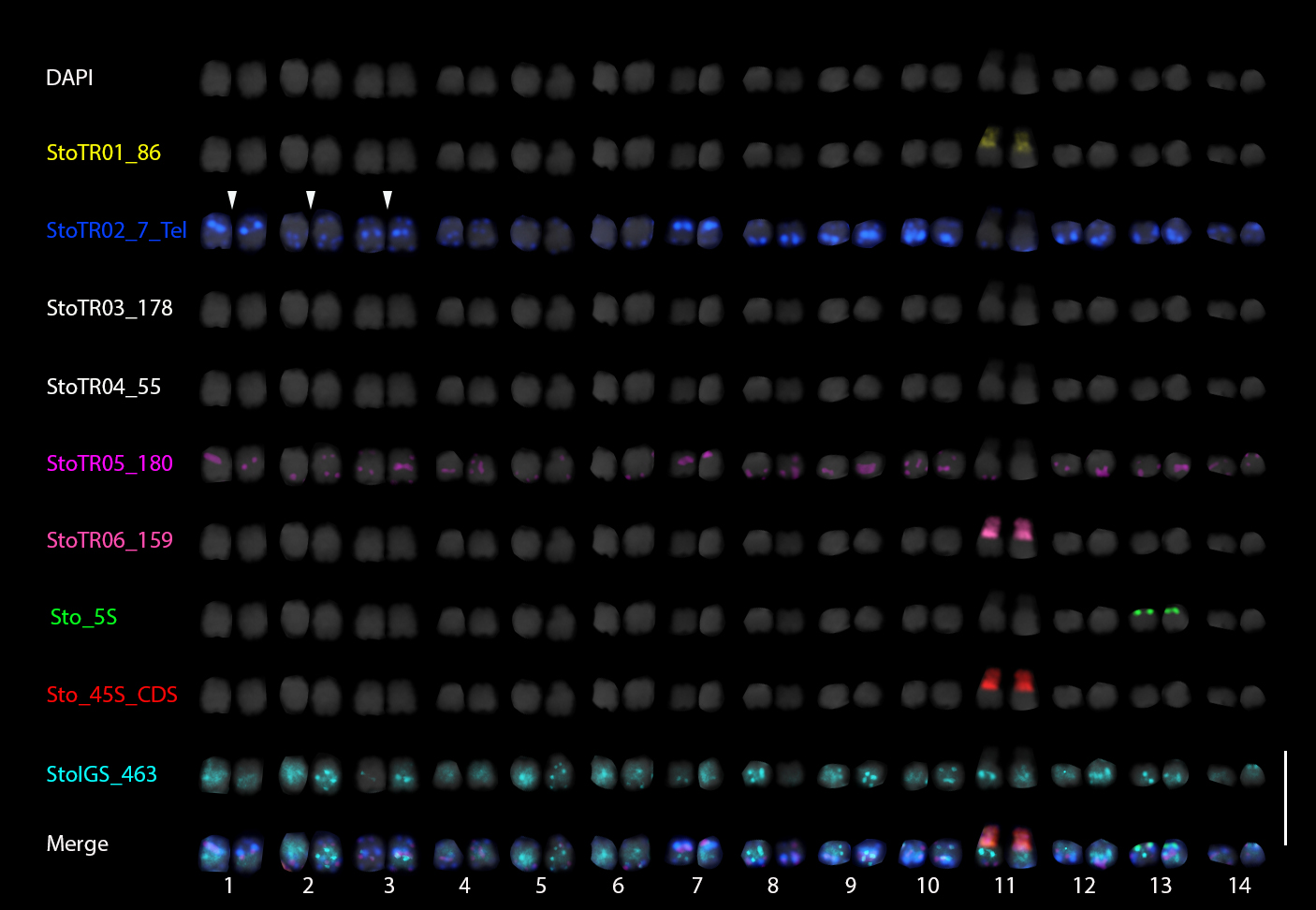
**

**Fig. S11** FISH karyograms of *S. corymbosa* with nine TRs probes. The white arrows indicate the ITR signals on the chromosomes 1, 2, and 3. Scale bar = 10 µm


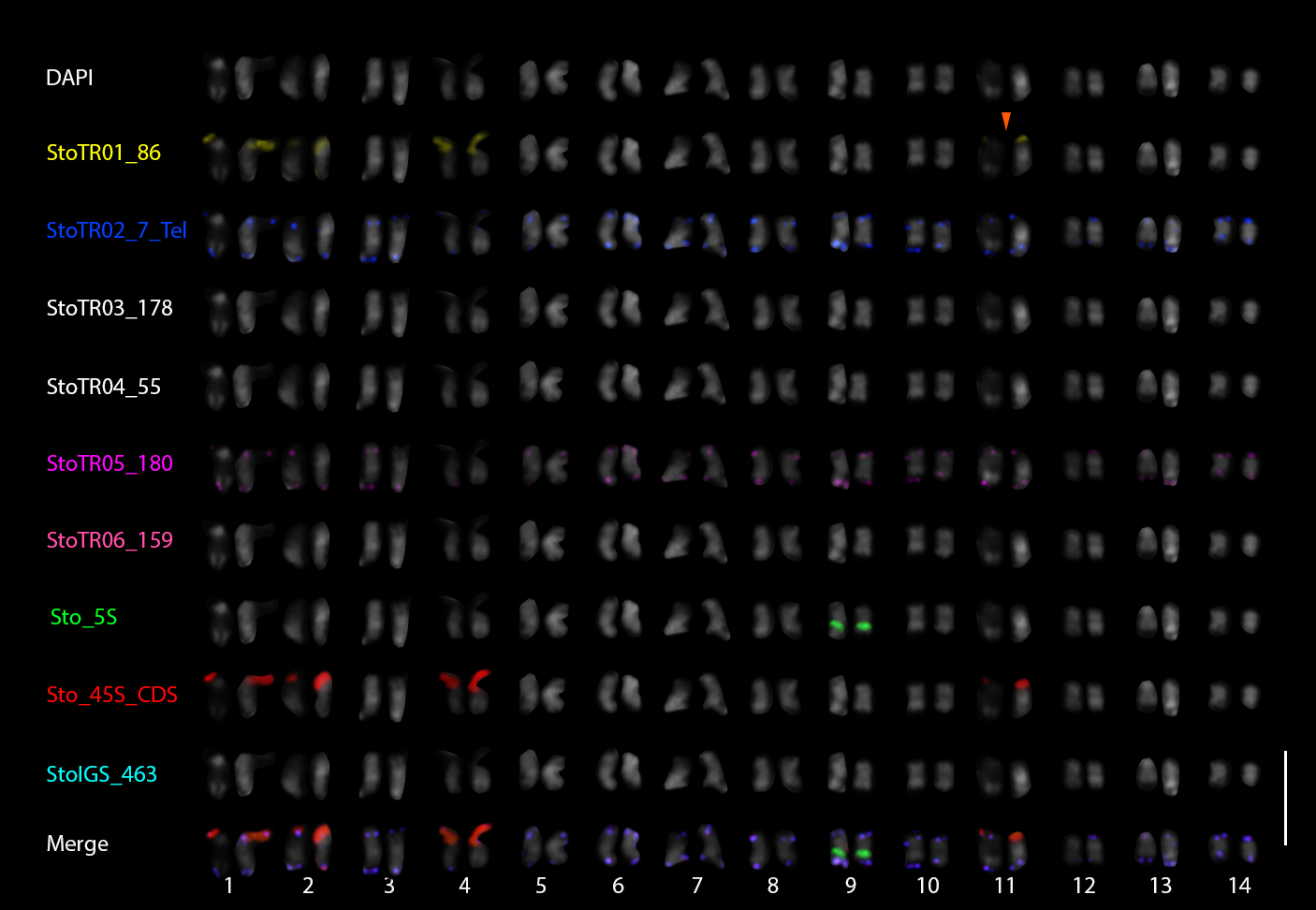


**Fig. S12** FISH karyograms of *S. didymobotrya* with nine TRs probes. The yellow arrows show the weak signal of StoTR01_86. Scale bar = 10 µm

*
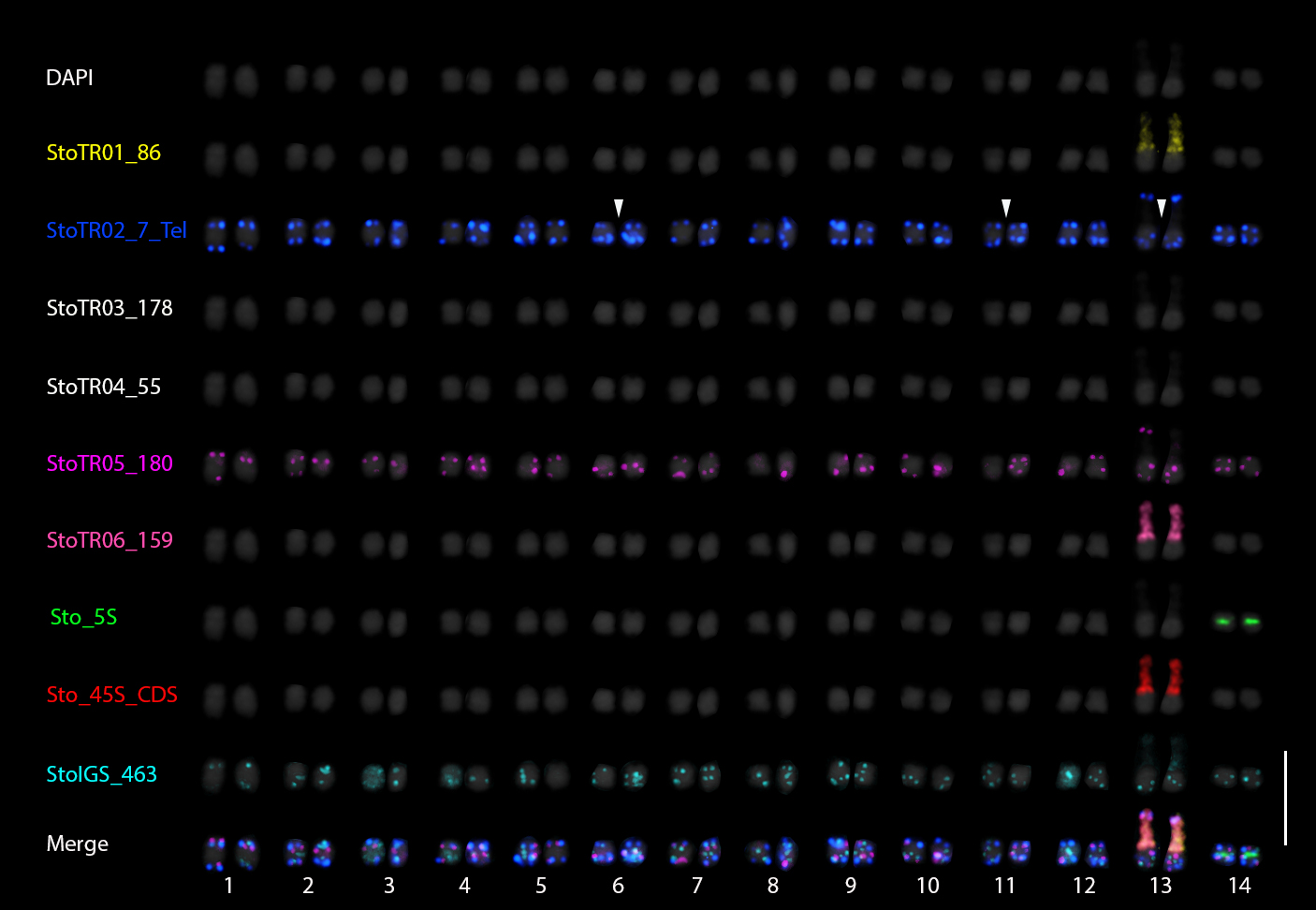
*

**Fig. S13** FISH karyograms of *S. floribunda* with nine TRs probes. The white arrows indicate the ITR signals on the chromosomes 6, 11, and 13. Scale bar = 10 µm

*
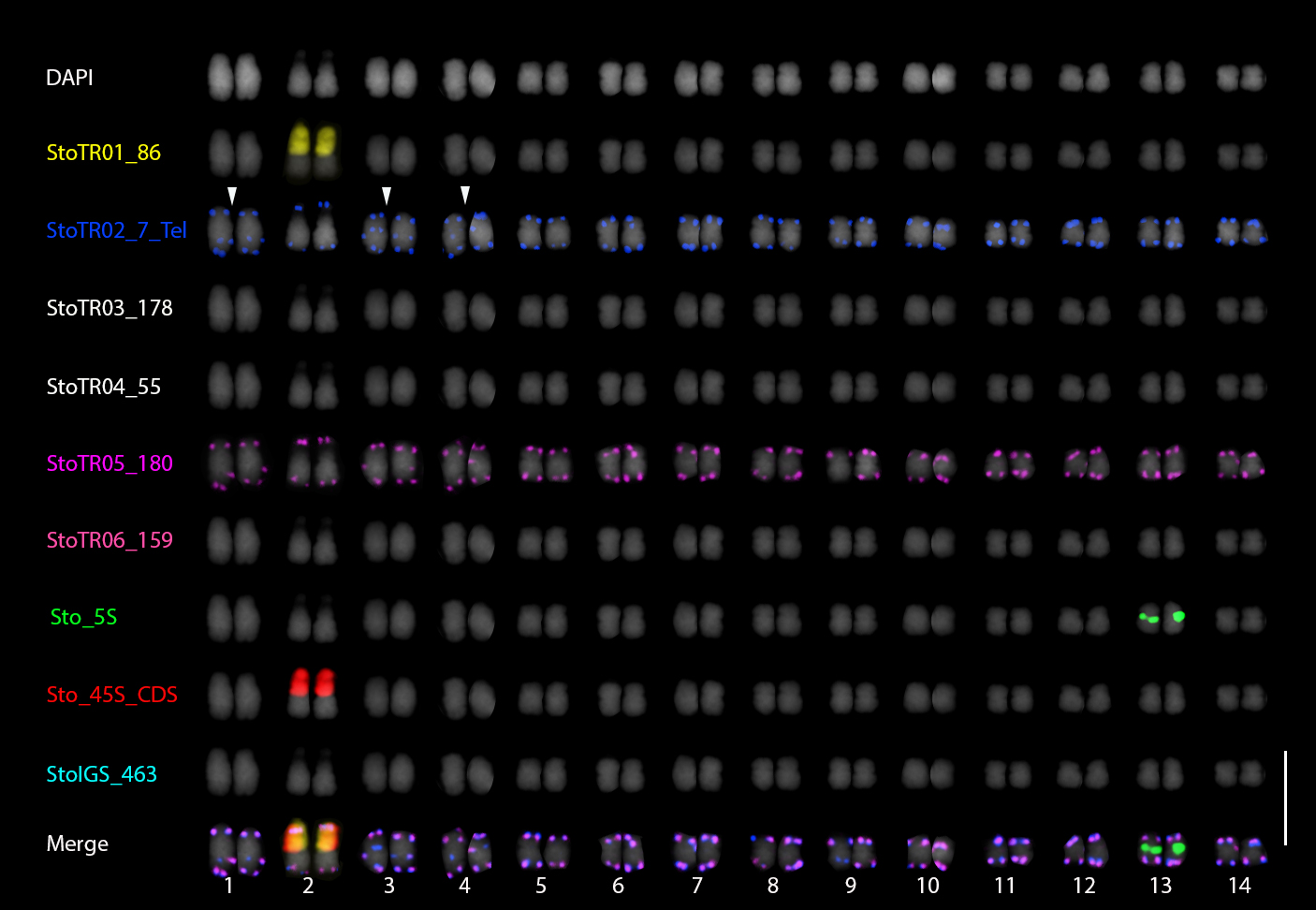
*

**Fig. S14** FISH karyograms of *S. occidentalis* with nine TRs probes. The white arrows indicate the ITR signals on the chromosomes 1, 3, and 4. Scale bar = 10 µm


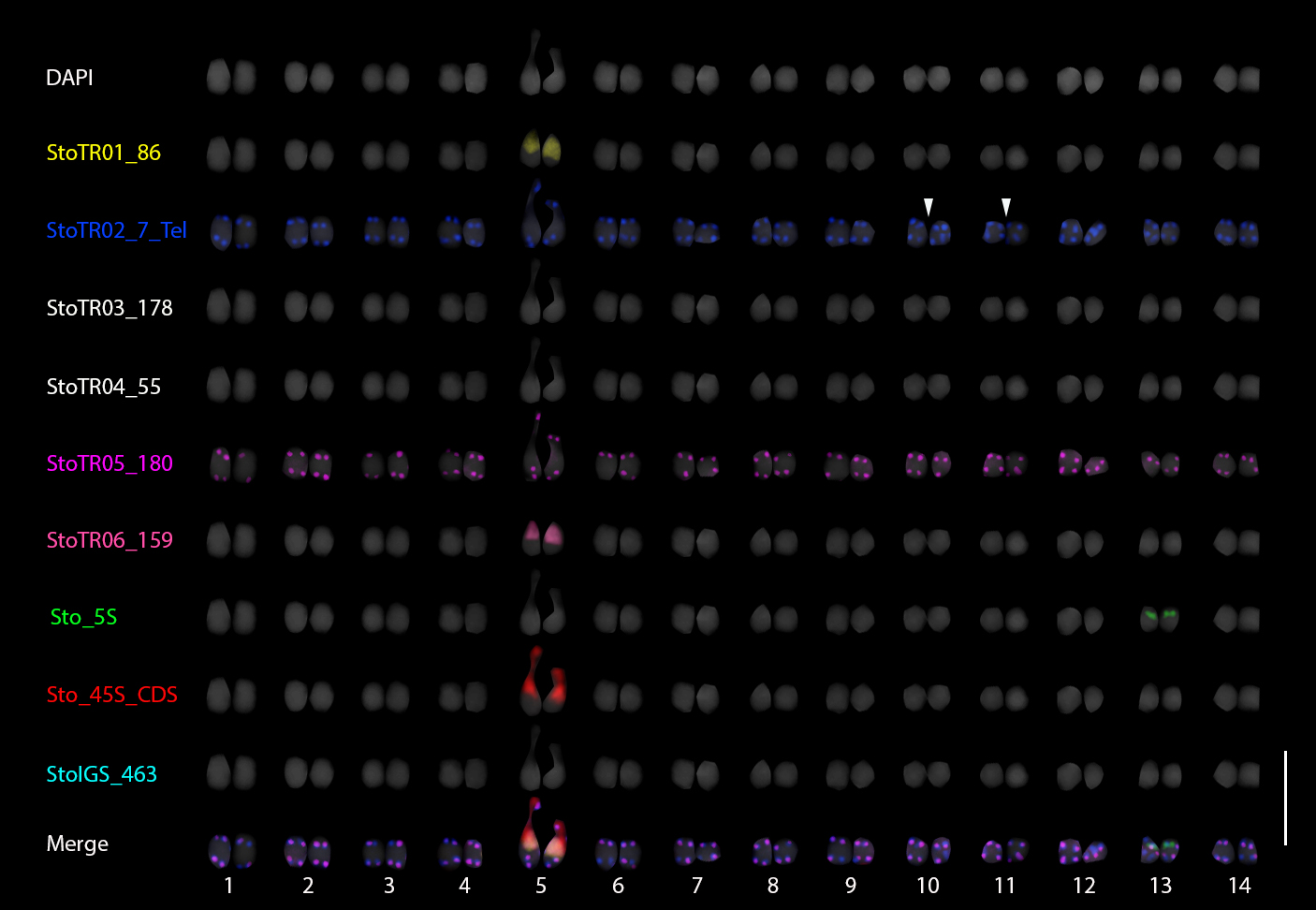


**Fig. S15** FISH karyograms of *S. multiglandulosa* with nine TRs probes. The white arrows indicate the ITR signals on the chromosomes 10, and 11. Scale bar = 10 µm

*
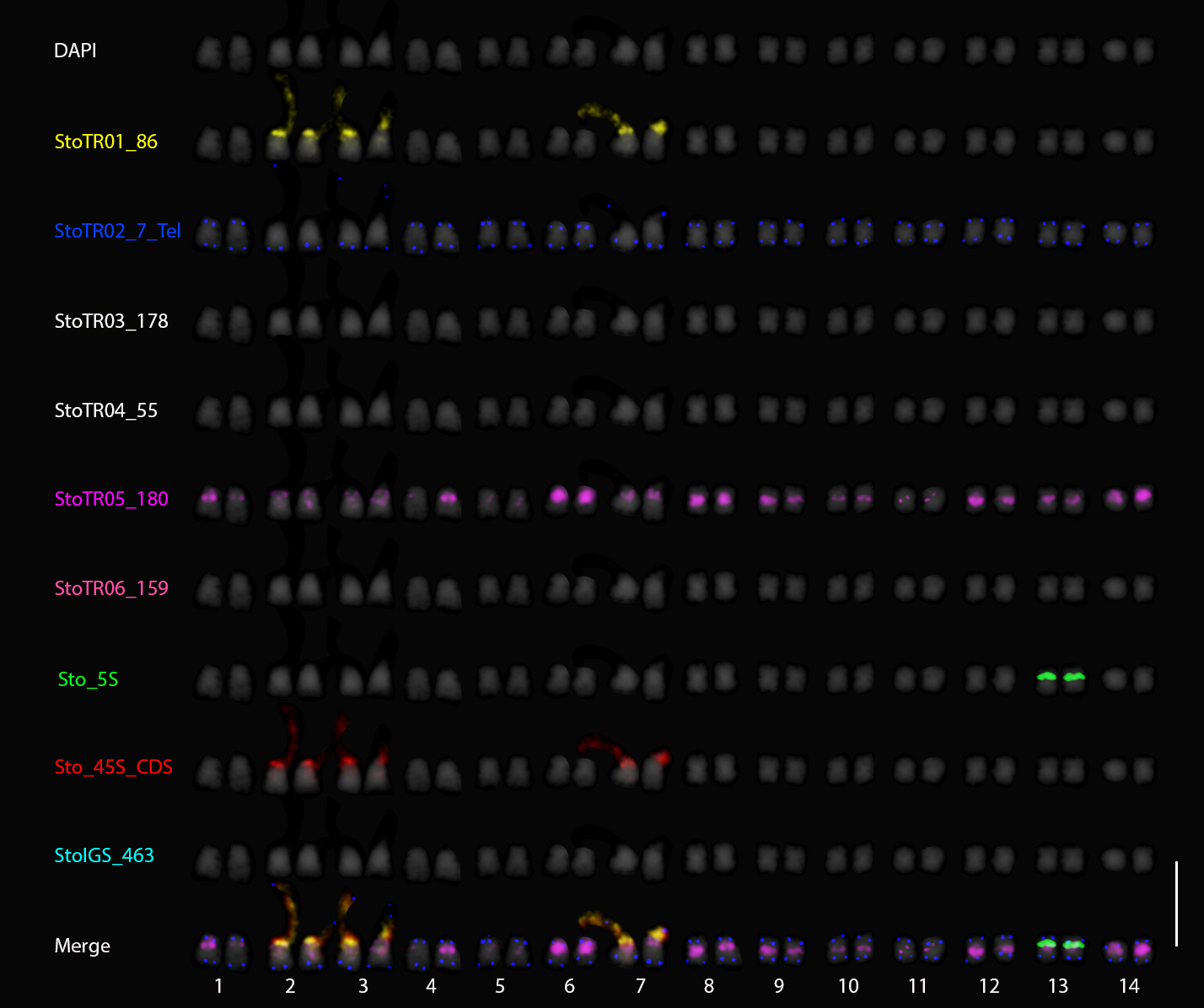
*

**Fig. S16** FISH karyograms of *S. sulfurea* with nine TRs probes. Scale bar = 10 µm

*
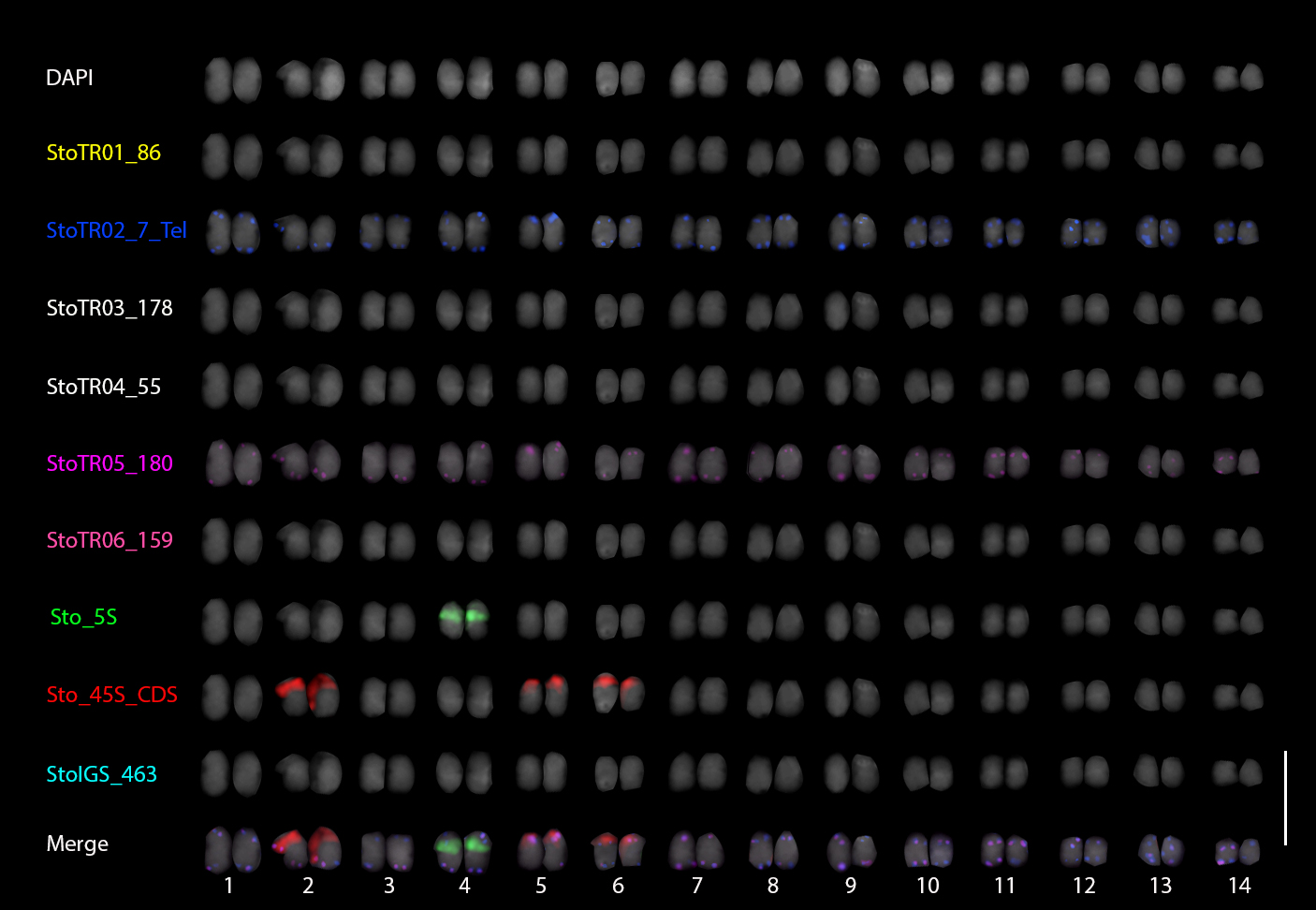
*

**Fig. S17** FISH karyograms of *S. siamea* with nine TRs probes. Scale bar = 10 µm
